# Treatment of *Leishmania infantum* Infections in Dogs

**DOI:** 10.3390/microorganisms13051018

**Published:** 2025-04-29

**Authors:** Melanie Kaempfle, Katrin Hartmann, Michèle Bergmann

**Affiliations:** LMU Small Animal Clinic, Centre for Clinical Veterinary Medicine, LMU Munich, 80539 Munich, Germany; m.kaempfle@medizinische-kleintierklinik.de (M.K.);

**Keywords:** canine leishmaniosis, therapy, meglumine antimoniate, miltefosine, allopurinol, leishmanicidal, leishmanistatic, antiparasitic drugs

## Abstract

Dogs are reservoir hosts of the zoonotic parasite *Leishmania infantum*, the causative agent of canine leishmaniosis. Antiparasitic drugs that are commonly used in dogs include allopurinol, miltefosine, and meglumine antimoniate. Treatment success is characterized by an improvement of disease signs, reduction in parasite load, as well as prevention of relapse. However, despite treatment, infections in dogs can usually not be cleared and often lead to (recurrent) signs of disease. Since most of the drugs used in dogs are also applied in human medicine, the prevention of treatment-induced drug-resistant *Leishmania* strains is a major one-health concern. This review article provides an overview of current treatment options for *Leishmania*-infected dogs with allopurinol, meglumine antimoniate, and miltefosine, related adverse effects, and drug resistance potential.

## 1. Introduction

**Figure 1:**
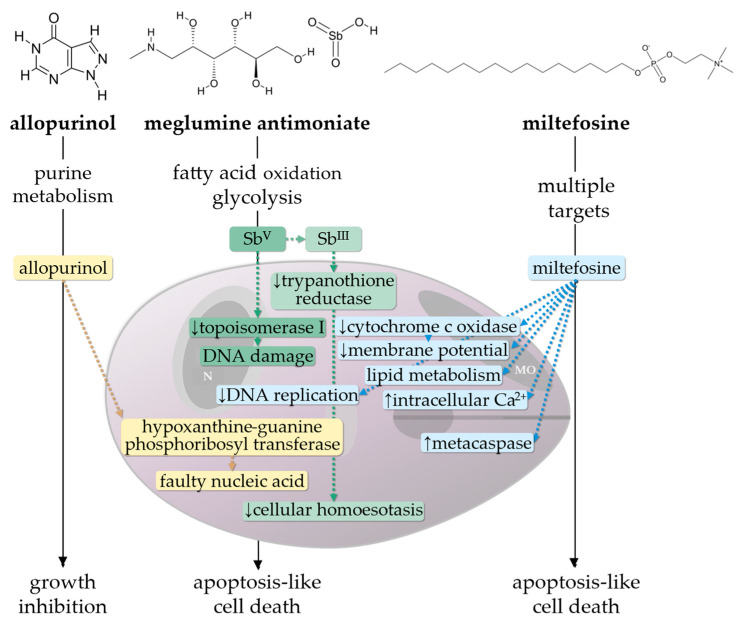
Effect of allopurinol, meglumine antimoniate and miltefosine on intracellular *Leishmania* amastigotes [22,23,24]. The hypoxanthine analogue allopurinol interferes with the parasitic purine salvage pathway; as a substrate of the enzyme hypoxanthine-guanine phosphoribosyl transferase, it is phosphorylated and integrated in nucleic acid, leading to growth inhibition [22,25]. Pentavalent antimony (Sb^V^), which is the active ingredient in meglumine antimoniate, is assumed to inhibit glycolysis, fatty acid oxidation, and enzymatic activity of DNA topoisomerase I, but exact antileishmanial mechanisms remain partially unclear. Reduction of Sb^V^ to the more toxic trivalent form (Sb^III^), which has high affinity for thiol-containing proteins, causes inhibition of the enzymatic activity of trypanothione reductase. Redox state and transcription regulation are disturbed, resulting in oxidative stress and cell death [26,27]. Miltefosine reduces the mitochondrial membrane potential and inhibits cytochrome c oxidase activity, which leads to an increased production of reactive oxygen species. Additionally, an effect on membranous Ca^2+^ channels and parasitic organelles (acidosomes) causes disturbance in Ca^2+^ homeostasis. Miltefosine further leads to an enhancement of metacaspases and inhibition of the parasites’ DNA synthesis and might influence lipid metabolism, for which exact mechanisms remain partially unclear [28,29,30]. MO, mitochondrion; N, nucleus.

Protozoan parasites of the species *Leishmania* (*L.*) *infantum* (syn. *L. chagasi*) are the causing agents of canine leishmaniosis, an infectious disease that affects approximately 2.5 million dogs in Europe [1]. The disease is endemic in different areas all over the world (e.g., Southern Europe, Northern Africa, the Middle East) and linked to the geographical distribution of phlebotomine sandflies (*Phlebotomus* spp. and *Lutzomyia* spp.) which serve as the parasites’ vectors [2]. Climate changes cause the development of new habitats for the sandfly vector and a northern spread of the disease in Europe [3,4,5]. Dogs are reservoir hosts and thus play a central role within the transmission cycle of *L. infantum*, which can also infect humans and other mammals [6,7]. After the blood meal of infected female phlebotomine sandflies, phagocytic cells of the skin take up *Leishmania* that have been inoculated by the vector [8]. The biphasic parasites convert from an extracellular, flagellated, promastigote form to an intracellular amastigote form, proliferate, and infect further host cells. If dispersal of the parasites is not controlled by the dog’s immune system at the timepoint when infection is limited to the skin, the parasites spread via the lymphatic system and blood through the reticuloendothelial system, thereby establishing a systemic infection [9,10]. Not every infected dog develops signs of the disease; an effective Th1-mediated cellular immune response is considered to prevent the disease [11]. In contrast, an exuberant, Th2-mediated, humoral immune response predominates in diseased dogs [10]. The activation of B-cells and production of large amounts of gamma globulins contribute to the formation of circulating immune complexes (CIC) composed by *Leishmania* antigens and anti-*Leishmania* immunoglobulins (IgG, IgM, IgA) that can cause glomerulonephritis, vasculitis, polyarthritis, and/or uveitis [12,13]. Once infected, dogs are thought to harbor the parasites for their entire lives, regardless of the development of clinical signs [14]. A reliable, long-term parasitological cure can usually not be achieved, despite anti-*Leishmania* treatment. However, treatment can lead to (1) an improvement of disease signs and/or prevention of disease relapse, (2) a decrease in parasite load, (3) a reduction in transmission rates among dogs and humans by reducing sandfly infectivity, and (4) prolongation of survival time [15,16,17,18]. Furthermore, the treatment of infected dogs is important due to one-health concerns; since the majority of drugs are used in the treatment of *Leishmania* disease, in both human and veterinary medicine, the emergence and spread of (multi-) drug-resistant *Leishmania* strains must be prevented [2,19,20,21]. The aim of this review is to provide an overview of the current treatment options, considering specific leishmanistatic (allopurinol) and leishmanicidal (meglumine antimoniate and miltefosine) drugs for dogs (Figure 1). In addition to the antiparasitic drugs, symptomatic treatment, e.g., of proteinuria in case of glomerulonephritis, is crucial for survival and well-being, but will not be discussed in this article. Additionally, immunomodulatory drugs are commonly used in dogs with *Leishmania* infections but are also not the subject of this review.

## 2. Leishmanistatic Treatment

### 2.1. Allopurinol

Allopurinol (4-hydroxypyrazolo [3,4-d]pyrimidine) is the only leishmanistatic drug commonly used to treat canine *Leishmania* infections. A structural analogy to the purine derivative hypoxanthine leads to an integration into parasites’ nucleic acid and inhibits further replication of *Leishmania* (Figure 1) [31,32,33]. Allopurinol is considered one of the gold standard treatment options for dogs with *Leishmania* infections and is used alone or in combination with other (predominantly leishmanicidal) drugs [2]. In dogs, allopurinol is rapidly metabolized (approximate elimination half-life of 2 h) to oxypurinol, which has only minor effects on some *Leishmania* species (*L. mexicana*, *L. braziliensis*) in vitro [34,35,36]. This could be the reason that most authors recommend allopurinol treatment in dogs with *Leishmania* spp. infections with 10 mg/kg, q12h instead of 20 mg/kg, q24h [2,36]. Furthermore, an increased frequency of administration might reduce xanthine excretion peaks and thus xanthinuria, but studies on this are missing [37]. The simultaneous administration of food did not significantly influence pharmacokinetics of allopurinol in healthy dogs [36].

#### 2.1.1. Initial Treatment of Dogs with Manifest Leishmaniosis

Allopurinol monotherapy can lead to an improvement of disease signs in dogs with leishmaniosis (Table 1 and Table 2). In experimental studies, allopurinol monotherapy (20 mg/kg/day, PO, for 3 months) was initiated 7 months after *L. infantum* infection of six laboratory-bred beagle dogs. At this timepoint, all dogs showed mild clinical signs of the disease (e.g., lymphadenopathy, splenomegaly, mild cutaneous signs, conjunctivitis) [38,39]. The median albumin/globulin (a/g) ratio, white blood cell count, hematocrit, and platelet count were below the reference range and/or differed significantly from that of two uninfected control dogs. With allopurinol treatment, the clinical signs disappeared, and laboratory alterations normalized. A significant decrease in positive acute phase protein levels (canine C-reactive protein (CRP), haptoglobin (HP), serum amyloid A (SAA)), compared to pre-treatment levels, was recorded 1 month after treatment onset and parallelled the improvement of clinical signs. However, treatment with allopurinol neither led to a significant reduction in immunoglobulin (IgG, IgM) levels, nor to complete parasite clearance. Nevertheless, *Leishmania* loads measured by qPCR at the end of treatment and 7 and 14 months later in spleen aspirates were significantly lower than before treatment [38,39].

An improvement of disease signs in response to allopurinol treatment was also observed in dogs from different field studies in non-endemic areas, where re-infection is unlikely to occur. In a study in Switzerland, different clinical signs of the disease (skin lesions, arthritis with lameness, reduced general condition) were present in 22/31 dogs at the timepoint of inclusion. Allopurinol treatment (10–15 mg/kg, q12h, PO, 2–24 months) led to a complete remission of skin lesions in 11/12 dogs (after 1–5 months), arthritis-related lameness in 5/5 dogs (after 2–3 months), and an improved general condition (weight loss and apathy) in 4/5 dogs (after 2 months). In 14/23 dogs, initially low a/g ratios normalized within 1–16 months. Following treatment, azotemia resolved in 1/3 dogs within 3 months and deteriorated in the other two dogs during the observation period (euthanasia after 20 and 22 months). Antibodies determined by an immunofluorescence antibody test (IFAT) decreased by at least three titer steps in 10/31 dogs within 5–20 months; 7/27 dogs turned antibody-negative in an enzyme-linked immunosorbent assay (ELISA) within 2–25 months. Allopurinol treatment was discontinued in 6/31 dogs after 7–22 months and no dog experienced disease relapse [40]. In another study conducted in Switzerland, the clinical efficacy of allopurinol (10 mg/kg/day, PO, 2–24 months) was investigated in ten dogs infected with *L. infantum*. Within 2–6 months, remission of clinical signs was achieved in 9/10 dogs. None of the dogs relapsed during continuous treatment application. When allopurinol was withdrawn in four out of the nine dogs that reached clinical remission, signs (lymphadenopathy, skin lesions) re-appeared in three out of four dogs after 2–4 weeks. Allopurinol was re-started in two of the three relapsed dogs and they improved again. The presence of *Leishmania* was proven even in clinically cured dogs by PCR of blood samples (*n* = 4) and/or cultivation or PCR of lymph node samples (*n* = 8). Since parasitic DNA in blood could maintain sandfly infectivity, the authors did not recommend the single use of allopurinol treatment (especially in endemic areas) at the applied dose (10 mg/kg/day, PO) [41]. In a study conducted in Germany on 16 dogs with leishmaniosis, clinical improvement after allopurinol monotherapy (10 mg/kg, q12h, PO) was observed in three out of seven dogs, but not in the remaining four dogs within 3–5 weeks, which needed additional leishmanicidal (meglumine antimoniate) treatment [42]. In a retrospective cohort study in the Netherlands, allopurinol monotherapy (minimum 20 mg/kg/day, PO) was initiated in 46 dogs with leishmaniosis and given for at least 3 months. In 30 dogs, allopurinol led to the complete remission of clinical signs. The remaining 16/46 dogs needed additional leishmanicidal treatment after a median time of 3.5 months. The overall survival time of all dogs was 6.4 years [43]. Results of another retrospective study on dogs with leishmaniosis (*n* = 72) in Germany showed that allopurinol (even when given as monotherapy) was able to prolong survival time; treated dogs (*n* = 58) most commonly received allopurinol alone (*n* = 30) or in combination with meglumine antimoniate (*n* = 27) and had a significantly better prognosis than untreated dogs (survival benefit of 5.2 years). There was no significant difference in survival time between dogs treated with allopurinol alone and dogs that received allopurinol in combination with meglumine antimoniate [15].

In comparison to field studies from non-endemic areas, field studies in endemic areas often showed only partial improvement of disease signs in dogs following allopurinol monotherapy. The only randomized, blinded, and placebo-controlled clinical trial was conducted in Greece and evaluated the outcome of 37 dogs after treatment with allopurinol (10 mg/kg, q12h, PO, for 4 months). At the end of the 4-month observation period, there was a significant improvement in 11/13 clinical signs and a reduction in the frequency of 7/23 laboratory alterations. Complete clinical remission was only achieved in one dog. In 23 dogs, different clinical signs appeared (and were not prevented) despite treatment. Furthermore, allopurinol monotherapy was not able to counteract the deterioration of kidney function, since three dogs developed uremia and were withdrawn from the study. *Leishmania* antibodies decreased significantly but were still positive in 27/34 dogs at the end of the study; only 2/34 dogs became antibody-negative in both tests used (IFAT and ELISA). *Leishmania* loads in the bone marrow and lymph node decreased significantly (cytological examination), but PCR tests performed at the end of the study on bone marrow samples were (still) positive in all dogs, concluding that allopurinol monotherapy reduced parasite burden but did not lead to complete parasite clearance [44]. This is in line with some other studies, which were all conducted in endemic areas, where re-infection cannot be prevented completely. In a study from Brazil, allopurinol treatment (20 mg/kg, q24h, PO, for 90 days) led to an improvement of clinical signs in all eight dogs of the allopurinol treatment group. Parasite burden in bone marrow decreased within 63 days (below the detection limit in five out of eight dogs) after treatment initiation, but re-increased after allopurinol withdrawal; at the end of the 1-year observation period, *Leishmania* DNA was detected by qPCR in all dogs. After euthanasia, parasites were also detected in different other organs: in kidneys (four out of eight dogs), skin (three out of eight), liver (four out of eight), spleen (four out of eight) and lymph nodes (six out of eight). In six untreated control dogs, a worsening of clinical signs, accompanied by an insufficient parasite elimination in the bone marrow and different organs was observed [45]. In a study on six dogs in Italy, allopurinol (10 mg/kg, q12h, PO, for 90 days) led to a significant decrease in parasite load (quantitative PCR) only in skin samples, but not in the blood or lymph node [46]. So far, there is no study comparing blood and skin parasite load to sandfly infectivity after allopurinol monotherapy. However, in a xenodiagnostic study including five dogs treated with allopurinol only (10 mg/kg, q12h, PO, for 6 months), a significant reduction of parasites in the bone marrow and sandfly infectivity (*Phlebotomus perniciosus*) was proven [18].

The treatment success of allopurinol monotherapy in field studies conducted in endemic areas was also proven by the remission of laboratory alterations and a reduction of acute phase protein levels, which (as in several other diseases) tend to be high in dogs with manifest leishmaniosis [47]. In a clinical trial, six dogs infected with *L. infantum* were treated with allopurinol (15 mg/kg, q12h, PO) for 60 days; ceruloplasmin (CP) and CRP levels decreased significantly from day 30 (CP) and day 60 (CRP) onwards; clinical signs of the disease improved and disappeared from day 20 onwards [48]. In another study, 19 dogs with leishmaniosis were treated with allopurinol (20 mg/kg, q12h, PO) until they reached complete remission (clinical signs and laboratory alterations) during a 4 to 7-month observation period. Acute phase protein levels were determined before treatment, after remission of clinical signs, after remission of laboratory alterations, and at a follow-up appointment after treatment was withdrawn. All dogs reached clinical cure within 2 months of treatment onset. After treatment, significant differences were observed in the concentrations of hemoglobin and hematocrit (normalization in anemic dogs within 3 months), total serum protein, albumin, and a/g ratio. Between the onset and end of allopurinol treatment, CRP and HP levels decreased significantly. However, after the end of treatment, a significant re-increase in CRP levels was observed, but since there was no further follow-up of the dogs, it remains unclear whether this increase indicated re-emerging disease [49]. Further studies should investigate the use of acute phase protein levels, especially CRP, to monitor allopurinol treatment efficacy.

Little is known about the monitoring of treatment success by analyzing circulating T-cell populations; results of previous studies are inconclusive [50,51]. Dogs with signs of leishmaniosis were shown to have lower CD4+/CD8+ ratios and CD4+ levels, which might facilitate the parasites’ replication and dissemination and thus increase sandfly infectivity [52]. For allopurinol treatment (10 mg/kg, q12h, PO for 18 months), an effect on circulating T-cell populations was shown in a study including 19 *L. infantum* infected dogs. The levels of circulating CD4+ and CD8+ cells of the treated dogs were compared to pretreatment levels and 16 healthy control dogs. After allopurinol treatment, the dogs had significantly higher CD4+ levels and CD4+/CD8+ ratios, as well as lower CD8+ levels than before treatment. However, compared to healthy dogs, the infected dogs (regardless of treatment) had significantly lower levels of circulating CD4+ T-cells [53].

To investigate the efficacy of initial treatment with allopurinol, further studies compared allopurinol monotherapy and its combination with leishmanicidals (Table 2). Commonly, a better and more reliable improvement of disease signs was observed following combination therapy. In a study in an endemic area in France, 96 dogs with leishmaniosis were monitored up to 6 years after assignment to three different groups and subsequent treatment either with (1) meglumine antimoniate in combination with allopurinol (*n* = 45), (2) meglumine antimoniate alone (*n* = 40), or (3) allopurinol alone (15 mg/kg, q12h, PO, for 1–20 months) (*n* = 11). Significant differences in the number of dogs that reached clinical cure were observed, with the best results in dogs of group 1 (combination allopurinol with meglumine antimoniate) and poor results in dogs from group 3 (sole allopurinol treatment), in which only two dogs reached clinical cure after 9 and 20 months; the worsening of physical conditions in the remaining nine dogs after 1 month led to termination of the study trial [54]. A further benefit of combined treatment was also proven in terms of the normalization of acute phase protein levels, with a significant decrease in group mean values of CRP and CP levels already from treatment day 10 onwards, compared to day 30 (CP) and 60 (CRP) in dogs treated with allopurinol only. The importance of these results, however, remains unclear, since an improvement of clinical signs was observed in both groups from day 20 onwards [48]. A better effect of combined treatment was also proven with regard to the prevention of disease relapses. In a retrospective case evaluation of 24 dogs with leishmaniosis in Italy, clinical signs improved in six out of six dogs that received allopurinol treatment (15 mg/kg, q12h, PO, for 12 months) within 2 months. However, four out of six dogs did not reach normalization of clinicopathological alterations during the 1-year treatment period and had disease relapses within 2–11 months after allopurinol withdrawal. In six out of six dogs of the group with additional meglumine antimoniate therapy, both clinical and clinicopathological improvements up to normalization were observed within 1–5 months after treatment onset, and no dog had disease relapse during the observation period. In dogs that received meglumine antimoniate as monotherapy, disease relapses were observed in 7/12 dogs during the 1-year study period (between 5–12 months) [55]. A significantly better effect on disease outcome (clinical score and normalization of clinicopathological abnormalities) was observed in dogs treated with a combination of allopurinol (10 mg/kg, q12h, PO, for 6 months) and meglumine antimoniate (35 mg/kg, q12h, SC, for 28 days) compared to dogs treated with each drug as monotherapy in a study conducted on 32 naturally infected dogs in Spain. A significant reduction in the number of dogs with detectable *Leishmania* spp. in bone marrow (cultivation) and dogs infective for sandflies was observed for all treatment regimens [18]. The outcome of a group of 15 allopurinol-treated dogs (20 mg/kg, q12h, PO, for 28 days) was compared to dogs that received allopurinol in combination with miltefosine or miltefosine monotherapy during a short-term observation period of 28 days. In dogs from all groups, clinical signs improved significantly. A significant increase in low a/g ratios was observed only in dogs that received combined treatment [56]. Allopurinol (used alone or in combination with miltefosine) was able to significantly reduce skin parasite loads [57].

Since, overall, a synergistic effect with better, more reliable and sustainable outcomes was proven for the combination of allopurinol with leishmanicidals (meglumine antimoniate or miltefosine), allopurinol monotherapy is currently only considered as a treatment option for dogs with mild clinical signs and negative or low positive antibody titers. In more severe cases, combined therapy is considered the gold standard [2].

#### 2.1.2. Maintenance Treatment of Dogs Infected with *L. infantum*

After its use in the initial treatment of diseased dogs (usually in combination with leishmanicidal treatment), allopurinol is commonly administered as a maintenance drug to prolong disease-free intervals and to prevent disease relapses, which can be observed especially once allopurinol is withdrawn [41,55,58]. Furthermore, maintenance treatment with allopurinol was shown to keep sandfly infectivity low in dogs after initial combined treatment [18]. However, since there are no controlled studies with an experimental design and/or without risk of re-infection, little is known about the ideal treatment length or intervals. In a study conducted in Spain, allopurinol (20 mg/kg/day, PO) was administered for 1 week per month to 15 dogs naturally infected with *L. infantum* after they reached clinical cure in response to combined treatment (meglumine antimoniate: 100 mg/kg/day, SC, for 20 days followed by a 10-day course after a 10–15-day resting period, and allopurinol: 30 mg/kg/day, PO, for 3 months). Clinical cure (without relapse) was maintained in the dogs during a 10 to 44-month follow-up period, while all 15 retrospectively evaluated control dogs (*L. infantum* infection; initial combined treatment) relapsed within 4.5–21 months [59]. In another study, the effect of allopurinol treatment (20 mg/kg, q24h, PO) for 1 week per month (April–November 1999) was investigated in 25 infected dogs without signs of the disease and compared to a placebo group. Bone marrow PCR was still positive in 18/19 allopurinol and 14/16 placebo treated dogs available for follow-up after 1 year, which is why the authors did not recommend this treatment regimen, especially in regard to the development of drug resistance [60]. Current recommendations comprise treatment with allopurinol for a minimum of 6 months and consideration of withdrawal only in cases of complete remission of clinical signs and laboratory alterations together with a marked decrease in antibodies (negative or borderline) [2,61]. Since many dogs do not reach this state, long-term (up to lifelong) allopurinol maintenance treatment is the consequence and (still) widespread common practice [62].

**Table 1:** Treatment of *Leishmania*-infected dogs with allopurinol in the last 25 years.

Design	Dosage	Length		Dogs		Control Intervals	Outcome				Adverse Effects	Ref.
		T	O	Incl.	Excl.		Clinical Signs	Laboratory Alterations	Parasite Detection			
									Indirect	**Direct**		
uncontrolled	**AL** 10 mg/kg /day	2–24 m	2–24 m	10	1 (renal failure)	every 1–2 m initially, then longer	remission within 2–6 m in 9/10 dogs, relapse after 2–4 w of drug withdrawal in 3/4 dogs	normalization of hct in 1/3 dogs within 4 m, glob in 3/10 dogs, alb in 5/8 dogs	persistent high IgG/IgG2 in 8/10 dogs, decrease in IgG1	pos. PCR/cultivation in LN of 8/9 and in PB of 4/9 dogs after clin. improvement	none	[41]
placebo- controlled randomized blinded	**AL** 10 mg/kg q12h	4 m	4 m	37	3 (renal failure)	every month	remission within 4 m in 1/34 dogs, reduction * of 11/13 signs	reduction * of 7/23 alterations	decrease * in IFAT titer/ELISA level, neg. in 2/34 dogs	decrease * in load (cytology) in BM and LN, persistent pos. BM PCR in all dogs	none	[44]
uncontrolled	**AL** 10 mg/kg q12h	90 d	90 d	6	--	0, 90 d	score reduction *	slight improvement in serum protein pattern, worsening in 2/6 dogs	no * change in IFAT titer; pos. in 6/6 dogs	decrease in load (PCR) in skin *, PB and LN, re-increase in 3 dogs after 90 d	n.a.	[46]
uncontrolled	**AL** 20 mg/kg q12h	until remission	4–7 m	19	--	every month	remission within 2 m	difference * in hb, hct (normalization in anemic dogs within 3 m), tp, alb, a/g, decrease * of CRP, HP, re-increase * of CRP after T	normalization of γ-glob within 6 m	n.a.	n.a.	[49]
controlled experimental	**AL** 20 mg/kg /day	3 m	17 m	6	1 (renal failure)	0, 3, 5, 7, 10, 17 m	remission within 1 m	normalization of hct, wbc, a/g	no * difference in IgG and IgM	decrease * in load (PCR) in spleen	n.a.	[39]
			14 m			0, 1, 2, 4, 5, 7, 11 m		decrease * of CRP, HP, SAA within 1 m				[38]
controlled	**AL** 10 mg/kg q12h	18 m	n.a.	19	--	before and after T	n.a.	increase* of CD4+ T-cells and CD4+/CD8+ ratio, decrease * of CD8+ T-cells	n.a.	n.a.	n.a.	[53]
uncontrolled	**AL** 10–15 mg/kg q12h	2–24 m	2–36 m	31 (9 w/o clin. signs)	3 (renal failure, ascites)	⌀ every 3 m	remission of skin lesion (11/12 dogs) within 1–5 m, lameness (5/5 dogs) within 2–3 m, reduced general condition (4/5 dogs) within 2 m	normalization of increased crea/urea in 1/3 dogs within 3 m and decreased a/g in 14/23 dogs within 1–16 m	decrease in IFAT titer of at least 3 steps in 10/31 dogs within 5–20 m, neg. ELISA in 7/27 dogs within 2–25 m	n.a.	xanthinuria	[40]
controlled randomized	**AL** 20 mg/kg q24h	90 d	360 d (euth.)	8	--	0, 63, 90, 180, 360 d	improvement	n.a.	n.a.	decrease in load (PCR) in BM within 63 d, then re-increase; all dogs pos. after 1 y; partially pos. in other organs after euth.	none	[45]
retrospective	**AL** at least 20 mg/kg /day	at least 3 m	several years	46	n.a.	3, 6 m, every 6 m	clinical remission in 30/46 dogs within 3 m, additional leishmanicidal T in 16/46 dogs (after 0–23 m), overall survival time 6.4 y		n.a.	n.a.	n.a.	[43]
placebo- controlled randomized blinded	**AL** 20 mg/kg q24h	1 w per month (8 m)	1 y	25 w/o signs	6 (lost to follow-up)	0, 1 y	persistent asymptomatic	n.a.	pos. IFAT in 1/19 initially neg. dogs	neg. BM PCR in 1/19 initially pos. dogs	n.a.	[60]

AL, allopurinol administered orally; alb, albumin; a/g, albumin/globulin ratio; BM, bone marrow; clin., clinical; controlled design, studies included at least 2 treatment groups or comparison of treated dogs to healthy/untreated dogs; crea, creatinine; CRP, C-reactive protein; d, day; ELISA, enzyme-linked immunosorbent assay; euth., euthanasia; Excl., excluded during observation period; glob, globulin; hb, hemoglobin, hct, hematocrit; HP, haptoglobin; IFAT, immunofluorescence antibody test; Ig, immunoglobulin; Incl., included for treatment; kg, kilograms; LN, lymph node; m, month; mg, milligrams; n.a., not applicable; neg., negative; O, observation; PB, peripheral blood; PCR, polymerase chain reaction; pos., positive; q12h, every 12 h; q24h, every 24 h; Ref, reference in text; SAA, serum amyloid A; T, treatment; tp, total protein; wbc, white blood cells; w, week; w/o, without; y, year; γ, gamma; ⌀, on average; *, statistically significant.

#### 2.1.3. Metaphylactic and Prophylactic Use

In non-endemic areas without the constant risk of (re-)infection, the potential of metaphylactic allopurinol use in dogs with *Leishmania* infections (acquired in endemic areas) which have not (yet) developed signs of the disease is discussed to prevent or stop progression to an overt disease [63]. In a study on such dogs (infected but no clinical signs; *n* = 9) which received allopurinol (10–15 mg/kg, q12h, PO, 2–24 months), no disease signs appeared during the observation period (2–36 months). However, since there was no (untreated) control group, it remains unclear whether allopurinol contributed to the lack of development of signs [40]. Recommendations for management are inconclusive. While some guidelines advise not to treat such dogs, others consider treatment, which might be important especially in non-endemic regions with expected sandfly occurrence in the future, to reduce parasite burden and thereby limit the spread [64,65].

In endemic areas, in which it is probable that nearly all dogs will be infected once in their life and a continuous infection stimulus is provided by the endemic occurrence of sandflies, the metaphylactic treatment of infected dogs without signs of the disease is usually not recommended and should, if at all, only be considered in dogs with high or increasing antibody titers [2,10,14,65].

The usefulness of prophylactic treatment with allopurinol (20 mg/kg, q24h, PO) for 1 week per month (April-November 1999) was evaluated in 26 non-infected dogs living in a highly endemic area in Greece and results were compared to 21 non-infected dogs receiving placebo [60]. After 1 year, infection was proven (antibody and/or PCR) in 6/15 allopurinol-treated dogs and in 1/7 placebo-treated dogs that were available for follow-up examinations; none of the dogs had signs of leishmaniosis. Due to a lack of efficacy and the risk of emergence and spread of resistant *Leishmania* strains in endemic countries, allopurinol was not recommended as a preventive measure against *Leishmania* infections by the authors [60].

**Table 2:** Treatment of *Leishmania*-infected dogs with allopurinol alone compared to combined treatment with meglumine antimoniate or miltefosine and meglumine antimoniate or miltefosine alone in the last 25 years.

Design	Dosage	Length		Dogs		Control Intervals	Outcome				Adverse Effects	Ref.
		T	O	Incl.	Excl.		Clinical Signs	Laboratory Alterations	Parasite Detection			
									Indirect	Direct		
controlled not randomized	**AL** 15 mg/kg q12h	until clin. remission (1–20 m)	9 m	11	9 (poor response)	0, 9 m	poor improvement (and excl.) in 9/11 dogs within 1 m, remission in 2/11 dogs within 9 and 20 m	n.a.	neg. IFAT in 1/11 dogs	n.a.	n.a.	[54]
	**MA** 200 mg/kg q24h at 2 d intervals SC	until clin. remission or decreased IFAT titer (3–6 m)	9–60 m	40	18 (renal failure, relapse)	0, 9 m	remission in 22/40 dogs, relapse (worsening condition) in 6/40 dogs	n.a.	neg. IFAT in 12/40 dogs	n.a	n.a.	
	**MA** 100 mg/kg q24h SC +**AL** 15 mg/kg q12h	1 m 9 m	9–60 m	45	8 (renal failure, relapse)	0, 9 m	remission in 37/45 dogs (more * dogs than in other groups), relapse (titer increase) in 5/45 dogs	n.a.	neg. IFAT in 6/45 dogs	n.a	n.a.	
controlled random allocation	**AL** 15 mg/kg q12h	60 d	60 d	6	--	0, 10, 30, 60 d	improvement (start after 20 d), all dogs in good health	decrease * of CRP after 60 d, CP after 30 d, no * difference in SAA and HP	(no *) decrease in γ-glob	n.a.	n.a.	[48]
	**MA** 100 mg/kg q24h SC +**AL** 15 mg/kg q12h	20 d 50 d	50 d	6	--	0, 10, 20, 35, 50 d	improvement (start after 20 d), all dogs in good health	decrease * of CRP, CP after 10 d, slight re-increase of CRP after 50 d, no * difference in SAA, HP	(no *) decrease in γ-glob	n.a.	n.a.	
controlled not randomized	**AL** 10 mg/kg q12h	180 d	180 d	9	4 (several reasons)	0, 60, 120, 180 d	score reduction *, less effective than combined T	improvement, less effective than combined T	n.a.	reduction * of pos. BM cultures (1/4 initially pos. dogs) and sandfly infectivity (0/3 initially infective dogs) after 180 d	n.a.	[18]
	**MA** 35 mg/kg q12h SC	28 d	180 d	11	2 (several reasons)	0, 60, 120, 180 d	score reduction *, less effective than combined T	improvement, less effective than combined T	n.a.	reduction * of pos. BM cultures (1/9 initially pos. dogs) and sandfly infectivity (1/6 initially infective dogs) after 180 d	n.a.	
	**MA** 35 mg/kg q12h SC +**AL** 10 mg/kg q12h	28 d 180 d	180 d	12	--	0, 60, 120, 180 d	score reduction *, more effective * than other T	improvement, more effective * than other T	n.a.	reduction * of pos. BM cultures (3/8 initially pos. dogs) and sandfly infectivity (0/8 initially infective dogs) after 180 d	n.a.	
retrospective	**AL** n.a.	n.a.	n.a.	30	--	n.a.	longer * survival in dogs of all 3 groups compared to	identification of proteinuria,	n.a.	n.a.	n.a.	[15]
	**MA** n.a.	n.a.	n.a.	1	--	n.a	untreated dogs (*n* = 14) (survival benefit of 1900 d), w/o difference * between	hypoalbuminemia, renal azotemia and lymphopenia as prognostic	n.a.	n.a.	n.a.	
	**MA + AL** n.a.	n.a.	n.a.	27	--	n.a.	dogs treated with AL and MA + AL	parameters in all treated dogs	n.a.	n.a.	n.a.	
retrospective	**AL** 15 mg/kg q12h	12 m	12 m at least	6	--	⌀ every 2 m	improvement in 6/6 dogs within 2 m (follow-up data beyond O: relapse after 2–11 m in 4/6 dogs)	remission in 2/6 dogs, persistent abnormal serum protein pattern in 4/6 dogs (relapse after O)	n.a.	pos. LN cytology during O in most available samples (no more detail)	n.a.	[55]
	**MA** 50 mg/kg q12h SC	until lab. remission	12 m at least	6	--	⌀ every 2 m	remission in 5/6 dogs within 1 m, in 1/6 within 2–3 m, relapse in 3/6 dogs after 7–11 m	remission in 6/6 dogs within 2–4 m, lab. relapse prior to clin. relapse in 2/3 dogs	n.a.	pos. LN cytology in all 3/3 relapsed dogs	n.a.	
	**MA** 37.5 mg/kg q6h SC	21 d	12 m at least	6	--	⌀ every 2 m	improvement in 6/6 dogs within 1.5 m, relapse in 3/6 dogs after 11–12 m, worsening of 1/6 dogs with renal failure after 8 m	remission in 1/6 dogs, persistent abnormal serum protein pattern in 5/6 dogs, relapse in 1/6 dogs after 12 m	n.a.	neg. LN cytology after end of T, gradually pos. afterwards	n.a.	
	**MA** 50 mg/kg q12h SC with or followed +**AL** 15 mg/kg q12h	8 w/until lab. remission 6 m after MA	12 m at least	6	--	⌀ every 2 m	remission in 6/6 dogs within 1–3 m, no relapse	remission in 6/6 dogs within 2–5 m, no relapse	n.a.	neg. LN cytology (all of the few available samples)	n.a.	
controlled not randomized	**AL** 20 mg/kg q12h	28 d	29 d	15	--	0, 29 d	score reduction * of 38%, no remission, no * difference between T groups	increase * in low rbc, persistent normal wbc, crea, urea, ALT, persistent abnormal tp, glob, a/g, no * difference in IL-2, -6, -10 and IFN-γ levels	increase * in IFAT titer	n.a.	none	[56]
	**MI** 2 mg/kg q24h	28 d	29 d	15	--	0, 29 d	score reduction * of 16%, no remission, no * difference between T groups	no * increase in low rbc, persistent normal wbc, crea, urea, ALT, persistent abnormal tp, glob, a/g, no * difference in IL-2, -6, -10 and IFN-γ levels	no * increase in IFAT titer	n.a.	n.a.	
	**MI** 2 mg/kg q24h +**AL** 20 mg/kg q12h	28 d	29 d	15	--	0, 29 d	score reduction * of 35%, no remission, no * difference between T groups	increase * in low rbc and a/g, persistent normal wbc, crea, urea, ALT, persistent abnormal tp, glob, a/g, no * difference in IL-2, -6, -10 and IFN-γ levels	no * increase in IFAT titer	n.a.	none for AL	
controlled not randomized	**AL** 20 mg/kg q12h	28 d	29 d	15	--	0, 29 d	score reduction * of 58%, remission in 1/15 dogs, similar efficacy of all T	n.a.	no * decrease in IFAT titer	decrease * in load (PCR) in skin; pos. in 15/15 dogs	none	[57]
	**MI** 2 mg/kg q24h	28 d	29 d	15	--	0, 29 d	score reduction * of 37%, similar efficacy of all T	n.a.	no * decrease in IFAT titer	decrease in load (PCR) in skin; pos. in 15/15 dogs	n.a.	
	**MI** 2 mg/kg q24h +**AL** 20 mg/kg q12h	28 d	29 d	15	--	0, 29 d	score reduction * of 53%, similar efficacy of all T	n.a.	no * decrease in IFAT titer	decrease * in load (PCR) in skin; pos. in 15/15 dogs	none for AL	

AL, allopurinol administered orally; ALT, alanine aminotransferase; a/g, albumin/globulin ratio; BM, bone marrow; clin., clinical; CP, ceruloplasmin; controlled design, studies included at least 2 treatment groups or comparison of treated dogs to healthy/untreated dogs; crea, creatinine; CRP, C-reactive protein; d, day; Excl.; excluded during observation period; glob, globulin; HP, haptoglobin; IFAT, immunofluorescence antibody test; IFN, interferon; IL, interleukin; Incl., included for treatment; kg, kilograms; lab., laboratory; LN, lymph node; m, month; MA, meglumine antimoniate; mg, milligrams; MI, miltefosine administered orally; n.a., not applicable; neg., negative; O, observation; PCR, polymerase chain reaction; pos., positive; q6h, every 6 h; q12h, every 12 h; q24h; every 24 h; rbc, red blood cells; Ref, reference; SAA, serum amyloid A; SC, subcutaneous administration; T, treatment; tp, total protein; w, week; wbc, white blood cells; w/o, without; y, year; γ, gamma; ⌀, on average; *, statistically significant; dark green fields, meglumine antimoniate monotherapy; light green fields, meglumine antimoniate combined with allopurinol; dark blue fields, miltefosine monotherapy; light blue fields, miltefosine combined with allopurinol.

#### 2.1.4. Adverse Effects

The main adverse effect of allopurinol is the development of xanthinuria and xanthine stone formation [54,66]. In treated dogs, allopurinol and its active metabolite oxypurinol affect the purine catabolism by the inhibition of the enzyme xanthine oxidase. This subsequently results in an increased urinary excretion rate of xanthine which is poorly soluble in water. In a retrospective study on 320 dogs infected with *L. infantum* that received allopurinol treatment (7.7–18.8 mg/kg, q12h, PO), adverse urinary effects (xanthine crystals, renal mineralization, urolithiasis) were observed in 42/320 dogs; 22 dogs developed urolithiasis. The time between onset of treatment and diagnosis of adverse effects ranged between 3 weeks and 9 years (median 1 year) [66]. However, it remains unclear how the dogs were fed during this study, since diets low in purine are considered an essential preventive measure. Further studies with all dogs receiving the same low-purine diet are needed [67,68]. Although urinary adverse effects are common, there is no consensus about further allopurinol treatment in dogs that have developed xanthinuria and xanthine urolithiasis during allopurinol treatment [2,37]. Recent recommendations include the application of allopurinol (10 mg/kg) only once per day, a dose that did not promote urinary side effects in dogs (*n* = 18) that received allopurinol maintenance treatment for 6 years [69,70]. However, studies on the efficacy and potential to develop resistance in dogs treated with only half of the dose are needed.

Allopurinol and its active metabolite oxypurinol are mainly excreted through the kidneys. Direct nephrotoxic effects of allopurinol (metabolites) have not been observed in dogs. In a placebo-controlled study, it was investigated whether allopurinol treatment (10 mg/kg, q12h, PO, for 6 months) of dogs infected with *L. infantum* (*n* = 30) causes or worsens existing renal lesions, proteinuria, and/or glomerular filtration rates. Before allopurinol onset, 12/30 dogs had no proteinuria/no azotemia, 10/30 dogs had proteinuria, but no azotemia, and 8/30 dogs had proteinuria and azotemia. The placebo group consisted of ten dogs: five dogs without proteinuria/without azotemia, and five dogs with proteinuria only. Before treatment onset, renal biopsy revealed renal lesions (glomerular/tubulointerstitial) in all 40 dogs. In the allopurinol treatment group, 11/12 dogs remained non-proteinuric throughout the observation period, while UP/C ratios increased significantly in the respective (placebo-treated) control group and 2/5 dogs became proteinuric. A significant improvement of pre-existing proteinuria was observed after the end of allopurinol treatment; in 3/10 non-azotemic dogs, proteinuria resolved, while this was not the case in any of the proteinuric, non-azotemic control dogs. Allopurinol treatment led to a significant improvement of tubulointerstitial lesions in non-azotemic dogs. Glomerular lesions and filtration rate remained without significant change during allopurinol treatment. Of the dogs included with proteinuria and azotemia before allopurinol onset, three out of eight became uremic, whereas serum concentrations of urea and creatinine normalized in the remaining five dogs (but no significant decrease in creatinine and urea levels). However, since there were no azotemic control dogs, it remains unclear whether allopurinol had a positive or negative effect on uremia [71]. In the case of renal insufficiency, a reduced glomerular filtration rate can impair drug elimination and lead to a subsequent accumulation. Thus, a dose adjustment of allopurinol (e g. 5 mg/kg, q12h, PO) is suggested by different authors [72,73,74].

Although uncommon, mild gastrointestinal disorders (reduced appetite, anorexia, vomiting, and diarrhea) as well as pruritus were occasionally described in some studies and listed as adverse effect of allopurinol treatment [42,70].

#### 2.1.5. Drug Resistance Potential

Since *L. infantum* infections in dogs are considered chronic and usually cannot be eliminated, allopurinol treatment might evoke drug-resistant *Leishmania* strains; multiple treatment cycles, long-term, and low-dose treatment could be risk factors. Drug-resistant parasite strains can compromise individual treatment success and be transmitted to other dogs and humans. The World Health Organization (WHO) recommends treating canine leishmaniosis only with drugs neglected in human medicine, like allopurinol [75]. However, allopurinol can lead to a quick adaptation of *Leishmania* parasites in dogs. In an in vitro study, the susceptibility of amastigote (intracellular host) and promastigote (extracellular vector) stages of *L. infantum* strains isolated from two canine and three human patients from Portugal to different (commonly) used drugs (allopurinol, meglumine antimoniate, miltefosine, amphotericin B) was investigated. Dog 1 had been treated with allopurinol (at the timepoint of *Leishmania* strain isolation), dog 2 was untreated. In vitro, the promastigote and amastigote parasite stages of the isolated *Leishmania* strain from the treated dog showed low allopurinol susceptibility (highest half maximal inhibitory concentration, IC50), whereas promastigote parasite stages of the isolate from the untreated dog had high allopurinol susceptibility [76]. In a study including 19 naturally infected dogs in Israel, it was investigated whether an adaptation of *Leishmania* to allopurinol is related to disease relapse in dogs with allopurinol monotherapy. Parasites were isolated from (1) dogs with signs of the disease that had not received any treatment (*n* = 10), (2) dogs with successful treatment with allopurinol (*n* = 5) that did not have signs of the disease during 3 months before inclusion and (3) dogs with disease relapse after initial successful treatment with allopurinol and a disease-free interval of at least 3 months (*n* = 4). Allopurinol susceptibility in the promastigote stages of the isolates from dogs with current relapse was lower (significantly higher IC50 levels) than in both other groups. IC50 levels of promastigote parasite stages did not correlate significantly with the duration of allopurinol treatment and antibody levels. IC50 levels of intracellular amastigote parasite stages could not be determined, since allopurinol concentrations >300 µg/mL led to cytotoxic and adverse effects on host cells, but growth inhibition at 300 µg/mL allopurinol was significantly higher in non-treated dogs, which were only compared to relapsed dogs [33]. It was also shown that allopurinol resistance can be induced by increased drug pressure in vitro; stepwise increases in allopurinol concentration in cultured *Leishmania* isolates from untreated dogs with leishmaniosis first led to a reduction in parasite growth rate, which was followed by a significant (re-)increase (approximately from day 39 and 60). Significant (up to 20-fold) increases in IC50 levels were recorded [77].

### 2.2. Conclusions on Leishmanistatic Treatment

Allopurinol is an integral part of treatment in *Leishmania*-infected dogs and commonly applied at 10 mg/kg, q12h, PO together with a diet low in purine to counteract its main adverse effects: the formation of xanthine crystals and uroliths. It impedes the growth of *Leishmania* but has no direct toxic effects on the parasites and is usually not used to treat human leishmaniasis. In dogs with (severe) signs of the disease, allopurinol is commonly applied together with leishmanicidal agents and as maintenance treatment. Current guidelines suggest administration for at least 6–12 months and, thereafter, consideration of withdrawal in the case of complete remission and decrease in antibody titers [2]. For dogs infected with *Leishmania* but without or only mild signs of the disease, there is currently no agreement on the usefulness and necessity of allopurinol treatment. While it is not considered obligatory in endemic areas, allopurinol treatment in dogs from non-endemic areas (without permanent risk of re-infection) might be beneficial for disease control.

However, in vivo studies are needed to provide information on adaptation mechanisms of *Leishmania* in treated dogs and to obtain information about critical treatment lengths or dosing regimens.

**Figure 2:**
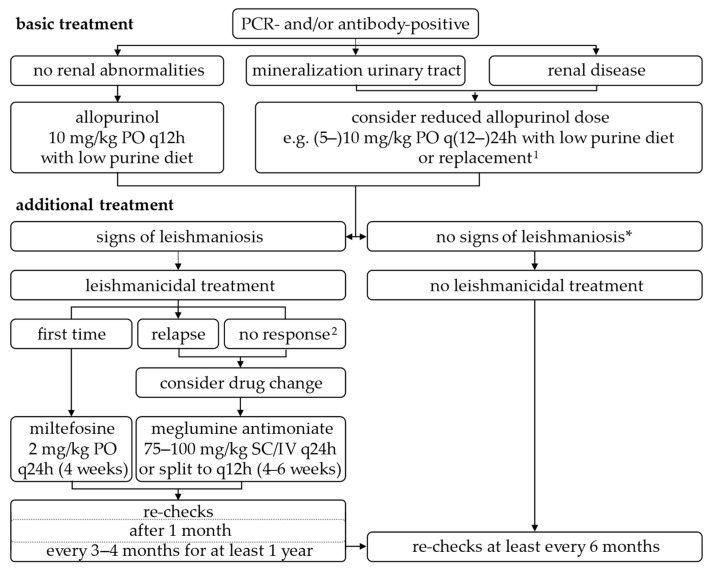
Therapeutic tree for dogs with *Leishmania* infections in non-endemic areas, according to authors’ opinion and in consideration of current recommendations [2,69,74]. * consideration of withdrawal after a minimum duration of 6 months, in case of complete remission and markedly decreased antibody level/no antibodies; ^1^ e.g., with domperidone or dietary nucleotides with active hexose correlated compound; ^2^ no improvement or worsening of clinical and/or laboratory signs within 4 weeks after end of treatment; IV; intravenous administration; kg, kilograms; mg, milligrams; PCR, polymerase chain reaction; PO, peroral administration; q12h, every 12 h; q24h, every 24 h; SC, subcutaneous administration.

Thus, the authors conclude that allopurinol should be administered (1) in dogs with (severe) signs of disease together with leishmanicidal agents, (2) as maintenance treatment following combination therapy with consideration of treatment withdrawal thereafter in the case of complete remission and decrease in antibody levels, and (3) as treatment option in antibody-positive dogs, in non-endemic areas, without signs of the disease. To ensure (long-term) treatment tolerability, regular ultrasound examination of the urinary tract and allopurinol dose reduction in case of adverse effects are considered essential (Figure 2, Table 3).

## 3. Leishmanicidal Treatment

### 3.1. Meglumine Antimoniate

Meglumine antimoniate is a complex of antimony (Sb (V)) and N-methyl-D-glucamine and is commonly used in the treatment of human and canine leishmaniosis [2,65,78]. For several decades, meglumine antimoniate has been considered a first-line treatment in dogs with clinical signs [79,80]. Administration typically comprises daily subcutaneous or intravenous injections at 75–100 mg/kg, q24h (or split q12h), for 28 consecutive days. Treatment prolongation for further 2–3 weeks in cases of inadequate improvement (not defined in more detail) is suggested by current guidelines [2]. The leishmanicidal activity of antimonial compounds is based on an inhibition of the parasites’ fatty acid oxidation and glycolysis, but the exact mechanisms are (still) poorly understood (Figure 1) [61,81]. In vitro, meglumine antimoniate shows immunomodulatory effects on monocytes and leucocytes, which are beneficial in the defense of intracellular parasites [82]. For dogs, meglumine antimoniate is available as an injectable solution for subcutaneous, intramuscular, or intravenous application. In a pharmacokinetic study on four healthy, non-infected dogs, subcutaneous injections (90 mg/kg) were associated with the longest drug half-life (approx. 2 h), longest time until peak serum levels were reached (approx. 3.5 h), and lowest mean peak values (126 µg/mL) compared to intramuscular (half-life: 42 min; peak time: 1.5–2 h; peak value: 161 µg/mL) and intravenous (half-life 21 min; peak time: 5 min; peak value 515 µg/mL) administration [83]. A longer half-life time (mean terminal elimination half-life of 618 min) after intravenous injection of meglumine antimoniate (100 mg/kg) was observed in another study on six healthy dogs. A comparison between intramuscular and subcutaneous application revealed no significant difference in peak time (intramuscular: 74 min; subcutaneous: 86 min) and peak values (intramuscular: 27 µg/mL; subcutaneous: 26 µg/mL). Due to the potentially higher risk of intramuscular injection-related adverse effects, the authors concluded that subcutaneous injections might be preferred [84]. In general, the results of the aforementioned pharmacokinetic studies demonstrate that injections several times a day (q8–12h) might be of advantage for continuous therapeutic plasma drug concentrations, which were observed in a study on six experimentally infected dogs treated with meglumine antimoniate at 75 mg/kg, q12h, SC, for 10 days [85]. In a randomized clinical trial on 41 naturally infected dogs in the Netherlands, no significant differences were observed in the parasitological and clinical outcomes between dogs after intravenous (*n* = 20) and subcutaneous (*n* = 21) injections of meglumine antimoniate (100 mg/kg, q24h, IV; 50 mg/kg, q12h, SC) for 21 days [86]. However, further studies are necessary to draw valid conclusions on an ideal treatment regimen [83,84].

#### 3.1.1. Initial Treatment

Monotherapy with meglumine antimoniate commonly leads to an improvement of disease signs. However, similarly to allopurinol monotherapy, parasite elimination is unlikely, and dogs are at risk of disease relapse (Table 2, Table 4 and Table 5). A (temporary) improvement of disease signs but insufficient parasite elimination following meglumine antimoniate monotherapy was observed in six experimentally *L. infantum*-infected dogs treated with meglumine antimoniate (75 mg/kg, q12h, SC, for two periods of 10 days, with 10 days resting period in between). Treatment was started (between 29 and 45 weeks after infection) if dogs had increased protein and gamma-globulin concentrations. At this timepoint, the dogs also had further signs of the disease (e.g., lymphadenopathy, thrombocytopenia, skin lesions). In the dogs that survived the treatment period (*n* = 5), remission of hyperproteinemia and other different disease signs were observed. *Leishmania* isolation was possible in lymph node aspirates between 1 day and 6 weeks after treatment until the end of the observation period (80–100 weeks after infection). Parasites in the buffy coat were detected in one out of five dogs only (28 weeks after treatment). Antibody levels (ELISA and dot-ELISA) decreased after treatment but remained above the cut-off and increased again after 3–10 months. In antibody Western blot analysis, a lower intensity of bands was observed after treatment. However, the reappearance of bands was observed in parallel with a re-increase in antibody levels measured by ELISA, and an increase in total protein and gamma-globulin fractions when dogs relapsed (all dogs between 6 and 10 months after treatment) [87].

In non-endemic areas, there are no recent studies on the use of meglumine antimoniate monotherapy, whereas some clinical trials were performed on naturally infected dogs in endemic areas. In a controlled, randomized multicenter study conducted in France and Spain, monotherapy with meglumine antimoniate (100 mg/kg, q24h, SC, for 28 days; *n* = 25 or 50 mg/kg, q12h, SC; *n* = 34, for 28 days) was shown to have good clinical and parasitological efficacy in the short term. A significant reduction of clinical scores (63.4%) and high parasitological efficacy (91.3% negative bone marrow cytology) was observed at the end of the 6-week study period, with less marked effects (33.3%) in the reduction of antibody titers and steady hematological and serum biochemistry values [88]. Remission of clinical signs, hematological, and urinary alterations within 60 days after initiation of meglumine antimoniate monotherapy (75 mg/kg, q12h, SC, for 21 days) was observed in another study, which included dogs with (*n* = 5) and without (*n* = 2) signs of the disease. However, disease relapse (uveitis, skin lesions) was observed in two out of five dogs 150 days after start of treatment. Parasites were not detected at any timepoint after treatment initiation in the bone marrow or lymph node cytology until day 180, when samples from four out of seven dogs were positive. After euthanasia, *Leishmania* parasites were observed in the spleen and/or liver tissue (cytology and cultivation) in five out of seven dogs [89]. In another study, parasite cultivation from the bone marrow was possible only in one of, initially, nine dogs 180 days after meglumine antimoniate monotherapy (35 mg/kg, q12h, for 28 days) and a significant reduction in sandfly infectivity was observed; while six out of nine dogs were infectious to sandflies before treatment, only one out of nine were infectious afterwards [18]. The decrease in *Leishmania* loads of different tissues following meglumine antimoniate monotherapy (100 mg/kg, q24h, SC, for 28 days, in two treatment courses with a treatment-free interval of 1 month) was investigated in another study with six dogs. Bone marrow, lymph node, blood, and hair samples were obtained at the timepoint of inclusion, after a first and a second treatment cycle, and during the following 4-month observation period. In general, the highest loads were measured by PCR in bone marrow and lymph nodes, and the lowest loads in blood samples. In two dogs, bone marrow parasite loads decreased progressively with each treatment cycle, whereas bone marrow PCR in two other dogs was positive only between both treatment cycles, and in the two remaining dogs, was negative throughout the whole study period. Parasite detection in the lymph node aspirates was possible in five out of six dogs before treatment onset; in four dogs, a decrease in load was observed following treatment, but there was no correlation with the course of bone marrow load. The PCR of hair samples was positive in all dogs with positive bone marrow PCR. Hair parasite load correlated with clinical score, *Leishmania* loads in other tissues, and IgG1 titer, which is why the authors concluded that it might be a useful tool to monitor treatment success. In one out of six dogs (in which parasites were not detected in any tissue after the two treatment cycles) clinical relapse occurred at the end of observation period and was accompanied by positive PCR results of bone marrow, lymph node, and hair samples, and an increase in IgG1 antibodies. In five out of six dogs, the clinical score was lower at the end than in the beginning of the study. Antibody titers remained stable throughout the study period in the majority of dogs and at the end of the observation period, parasite detection was possible in different tissues of five out of six dogs [90]. However, at least with the applied treatment schedules, meglumine antimoniate monotherapy was not able to prevent disease relapses. To obtain information on the ideal treatment regimen, the outcome of dogs treated either with a higher dose meglumine antimoniate (150 mg/kg/day) split into 37.5 mg/kg, q6h, SC, for 21 days (*n* = 6) or a lower dose (100 mg/kg/day) split into 50 mg/kg, q12h, SC, until clinicopathological recovery (*n* = 6) was retrospectively evaluated in another study. The higher dosing regimen did not result in higher efficacy, since only one dog achieved remission of laboratory alterations during the 1-year follow-up period and disease relapses were observed more frequently (four out of six dogs with relapse and another dog with development of kidney disease) than in the other group (three out of six dogs with relapse). Overall, the best outcome was observed in dogs treated with meglumine antimoniate together with or followed by allopurinol; in all six dogs of this group, the remission of clinical signs was observed within 1–3 months and remission of laboratory alterations within 2–5 months without deterioration or relapse in any dog until the end of the 1-year observation period [55]. These results are in accordance with current treatment guidelines, as a combined treatment with allopurinol and meglumine antimoniate is one of the gold standard treatment options, aiming to obtain long disease-free intervals, lower sandfly infectivity and shorten treatment length, thereby reducing the risk of adverse effects (Table 2, Table 4 and Table 5) [2,18,54,55]. In a controlled multicenter study conducted in different veterinary centers in Italy, Spain, and France, combined treatment with meglumine antimoniate (50 mg/kg, q12h, SC, for 28 days) and allopurinol (10 mg/kg, q12h, PO, for 7 months) was administered to 36 dogs. Within 3 months from treatment onset, a significant improvement in clinical signs (clinical and cutaneous score and weight loss) was observed. Parasite burden in bone marrow (qPCR) decreased significantly within the first month, whereas a significant decrease in antibody titers (IFAT) was observed after 3 months [91]. The efficacy of combined treatment was also evaluated in a study in Portugal, in which six dogs were treated with meglumine antimoniate (100 mg/kg, q24h (route of administration not given), for 4 weeks) together with and followed by allopurinol (10 mg/kg, q12h, PO, for at least 6 months). A remission of clinical signs was observed 3 months after treatment onset. Albumin/globulin ratios normalized within 2 months, followed by total protein and gamma-globulin levels 1 month later. All dogs turned antibody-negative (IFAT) within 3 months and parasites were not visualizable in lymph node and bone marrow smears (anymore). In addition, there was a tendency for the normalization of cytokine gene expression but persistence of a pro-inflammatory immune environment [92]. In another controlled study, the effect of combined treatment with meglumine antimoniate (50 mg/kg, q12h, SC, for 28 days) and allopurinol (10 mg/kg, q12h, PO, for 6 months) on acute phase protein levels was monitored for 3 months. Increased CRP levels were recorded in 7/12 dogs before treatment initiation. Treatment led to significant improvement of clinical signs and significant decrease in CRP levels (within 1 month), which (despite treatment) remained high in 2/12 dogs. Ferritin levels decreased significantly during treatment, while albumin levels and activity of the enzyme paraoxanase (PON-1), which are commonly low during acute phase reactions, increased significantly [93]. In a study in Italy, 18 naturally infected dogs were treated with meglumine antimoniate (100 mg/kg, q24h, SC) for 30 days together with and then followed by allopurinol (10 mg/kg/day, PO, for 2 years). Treatment led to a complete remission of clinical signs in 7/18 dogs within 3 months. In all dogs, there was an improvement of clinical signs within 1–6 months and a decrease in parasite loads in all tested tissues (skin, blood, lymph node). Durable remission of clinical signs that lasted from month 6 up to the end of the 30-month clinical observation period was achieved in 11/18 dogs, while in the remaining 7/18 dogs, disease relapse occurred between 9 and 24 months after combined treatment. In dogs with disease relapse, a concomitant re-increase in tissue parasite load was observed. After 2 years, *Leishmania* DNA was not detected (anymore) in the blood of 9/18 dogs, in the lymph node of 5/18 dogs, or in the skin of any dog by qPCR [94]. In another study, conducted in different veterinary centers in Spain, combined treatment with meglumine antimoniate (80–100 mg/kg, q24h, SC, for 1 month) and allopurinol (10 mg/kg, q12h, PO, for 1 year) was administered to 37 dogs with leishmaniosis. The dogs were examined before (*n* = 37), 30 days (*n* = 36), 180 days (*n* = 37), and 365 days (*n* = 29) after treatment initiation. *Leishmania* DNA was detected by PCR in blood samples of 23/36 dogs before treatment onset, with significantly higher loads than during the follow-up period. Between the beginning of the study and each timepoint afterwards, a significant decrease in antibody levels (ELISA) was observed; overall, five dogs turned antibody-negative during the study. Concomitantly, an improvement of disease signs (clinical and laboratory alterations) was observed; at the 6-month check-up, most of the dogs did not have any clinical signs. A remission of laboratory alterations was achieved in about 50% of the dogs after 6 months and in 65% after 12 months. Disease relapse occurred in three dogs with a concomitant increase in antibody levels and blood parasite loads. The use of blood parasite load to monitor treatment success, however, remains questionable, since increasing loads were also observed in eleven dogs without disease relapse (between 30 days and 1 year after treatment) [95]. In a study in Spain, no differences were observed in circulating T-cell populations between treated *Leishmania*-infected dogs (meglumine antimoniate and allopurinol) and healthy uninfected controls [50]. Since these findings are in contrast to those of other studies (see allopurinol section), the use of assessing the immune response for monitoring treatment success remains unclear and needs to be investigated in further studies [53].

Due to the chronic character of *Leishmania* infection with common relapses, the long-term outcomes of treated dogs are of special interest. To investigate long-term efficacy with a special focus on parasite clearance, nine dogs treated with meglumine antimoniate (100 mg/kg, q24h, SC, for 30 days) in combination with allopurinol (10 mg/kg, q24h, PO, for 6 years) were followed up retrospectively for 6 years. Significantly lower clinical scores were recorded at every timepoint after treatment initiation than before. During the observation period, only one out of nine dogs relapsed after 12 months (and received another leishmanicidal treatment cycle), whereas every other dog did not have any clinical signs at this timepoint. *Leishmania* antibodies (IFAT) decreased during treatment and the following observation period. *Leishmania* load, quantified by qPCR of lymph node samples, decreased significantly after 1 month and showed a progressive decline until month 9. After 3 months, the mean *Leishmania* load was 50-folds lower than before the start of treatment. An increase in mean parasite load was observed in month 12 due to an increase in parasite load of the relapsing dog. After 72 months, parasites were still detectable in eight out of nine dogs [70]. In another retrospective study conducted in Spain, clinical records of 23 dogs were evaluated. Dogs were followed up for 2–9 years after they were treated with meglumine antimoniate (100 mg/kg, q24h, SC, for 4 weeks) and allopurinol (10 mg/kg, q12h, PO, for at least 1 year). In total, 15/23 dogs received allopurinol during the whole period of follow-up. Complete remission of clinical signs was observed in 10/23 dogs after 1 month and in 20/23 dogs after 3 months (with only mild signs in the remaining 3 dogs); in 8/23 dogs, complete remission lasted until the end of the observation period. Furthermore, a restoration of serum protein levels (decrease in gamma globulins) was observed within 3 months. *Leishmania* antibody levels (ELISA) declined below the threshold in 4/23 dogs during the observation period. Disease relapse occurred in 3/23 dogs with a mixed pattern of signs (skin lesions, lymphadenopathy, anorexia, weight loss, dysproteinemia, and high *Leishmania* antibody levels) after more than 2 years from treatment onset. In one out of three of these dogs, allopurinol treatment was discontinued 1 year prior to the relapse; the other two dogs received continuous allopurinol treatment when relapse occurred. In 8/23 dogs that always had high antibody levels throughout the study, immune-mediated lesions emerged on day 145 in one, and after 1–2 years in the other seven dogs. Although meglumine antimoniate was applied more than once in 10/23 dogs, adverse effects were caused by allopurinol alone [96]. In conclusion, in these clinical trials, disease-free intervals after combined treatment ranged between 6 months and 2 years. However, since all studies were conducted in endemic areas, the risk of re-infection possibly influenced the results. Longitudinal studies of dogs living in non-endemic areas without risk of re-infection would (also) be valuable.

**Table 4:** Treatment of *Leishmania*-infected dogs with meglumine antimoniate alone and combined with allopurinol in the last 25 years.

Design	Dosage	Length		Dogs		Control Intervals	Outcome				Adverse Effects	Ref.
		T	O	Incl.	Excl.		Clinical Signs	Laboratory Alterations	Parasite Detection			
									Indirect	Direct		
experimental uncontrolled	**MA** 75 mg/kg q12h SC	2 × 10 d (10 d apart)	39–63 w	6	1 (hepatic, renal failure)	0, 31, 37, 60, 90 d, every 3 m	temporary remission, relapse in 5/5 dogs after 6‣10 m	normalization of tp and hematological alterations, (re-)increase in tp and γ-glob after 3 m	decrease in (dot-)ELISA and WB level, re-increase after 3–10 m	pos. LN cultivation between 1–42 d after T until end, PB pos. in 1 dog after 28 w	n.a.	[87]
uncontrolled	**MA** 75 mg/kg q12h SC	21 d	180 d (euth.)	7 (2/7 w/o signs)	--	every 30 d	remission in all dogs within 60 d, relapse in 2 dogs after 150 d	remission of hematological and urinary alterations in all dogs within 60 d	n.a.	neg. LN/BM cytology until 180 d, then pos. in 4/7 dogs, pos. spleen or liver cytology or cultivation after 180 d in 5/7 dogs	n.a.	[89]
uncontrolled	**MA** 100 mg/kg q24h SC	2 × 28 d (1 m apart)	210 d	6	--	0, 60, 120, 210 d	score reduction in 5/6 dogs, relapse in 1 dog at end O	n.a.	steady IFAT titers (IgG/-1/-2) in most dogs, increase in IgG1 in relapsed dog	pos. PCR of BM in 2/6, LN in 5/6, hair in 2/6, PB in 0/6 dogs (individual variations in load)	n.a.	[90]
controlled not randomized	**MA** 100 mg/kg q24h SC +**AL** 10 mg/kg q12h	30 d 1 y	1 y	28 (17/28 mild, 11/28 severe signs)	2 (death)	0, 1, 6, 12 m	remission in all dogs	no * difference in CD4/CD8+ ratio and CD8+, CD21+ to healthy dogs, difference * in CD4+ course between severely sick and healthy dogs	pos. *Leishmania* skin test in 11/11 initially neg. severely sick dogs	n.a.	n.a.	[50]
uncontrolled	**MA** 100 mg/kg q24h SC +**AL** 10 mg/kg /day	30 d 2 y	2 y at least	18	--	0, 1 m, then every 3 m	improvement in all dogs between 1 and 6 m, persistent remission until end in 11/18 dogs, relapse after 9–24 m in 7/18 dogs	n.a.	decrease in IFAT titer after 1 m	decrease in load (PCR) in PB, skin and LN within 1–3 m, persistent * LN load in 50% of cured dogs, re-increase in relapsed dogs, neg. PCR of PB in 9/18, LN in 5/18, skin in 18/18 dogs	none	[94]
uncontrolled	**MA** 80–100 mg/kg q24h SC +**AL** 10 mg/kg q12h	1 m 12 m	12 m	37	8 (lost to follow-up)	0, 30, 180, 365 d	improvement in all dogs within 30 d, remission within 6–12 m in most dogs, relapse in 1/37 dogs after 180 d	improvement in all dogs within 30 d, remission in 18/37 dogs after 180 d and 19/29 after 365 d, decrease * of UP/C, difference * in tp, alb, γ-glob, hct, hb, relapse in 1/37 dogs after 180 d	decrease * in ELISA level after 30 d, 6 m and 1 y, increase in relapsed dogs, neg. ELISA in 5 dogs during 1 y	decrease * in load (PCR) in PB within 30 d, increase in dogs with relapse and half AL dose, pos. PB PCR after 1 y in 9 previously neg. dogs	n.a.	[95]
retrospective	**MA** 100 mg/kg q24h SC +**AL** 10 mg/kg q12h	4 w at least 1 y	2–9 y	23	--	0, 1, 3, 6 m, every 6 m	remission within 3 m in 20/23 dogs; durable in 8/23, relapse in 3/23 (>2 y), emergence of immune-mediated lesions in 8/23 (within 2 y), repeated MA cycle(s) in 10/23 dogs	normalization of tp and γ-glob within 1–3 m	slow decrease in ELISA level, neg. in 4/23 dogs after 1 y	n.a.	urolithiasis	[96]

AL, allopurinol administered orally; alb, albumin; BM, bone marrow; controlled design, studies included at least 2 treatment groups or comparison of treated dogs to healthy/untreated dogs; d, day; ELISA, enzyme-linked immunosorbent assay; euth, euthanasia; Excl.; excluded during observation period; glob, globulin; hb, hemoglobin; hct, hematocrit; IFAT, immunofluorescence antibody test; IgG, immunoglobulin G; Incl., included for treatment; kg, kilograms; LN, lymph node; m, month; MA, meglumine antimoniate; mg, milligrams; n.a., not applicable; neg., negative; O, observation; PB, peripheral blood; PCR, polymerase chain reaction; pos., positive; q12h, every 12 h; q24h, every 24 h; Ref, reference; SC, subcutaneous administration; T, treatment; tp, total protein; UP/C, urine protein/creatinine ratio; w, week; WB, Western blot; w/o, without; y, year; γ, gamma; *, statistically significant; dark green fields, meglumine antimoniate monotherapy; light green fields, meglumine antimoniate combined with allopurinol.

A recent attempt to increase the efficacy of meglumine antimoniate treatment consisted of encapsulation in liposomes, which serve as drug delivery system. Liposomes are selectively taken up by cells of the mononuclear phagocytic system (MPS), of which many (e.g., macrophages of liver, spleen, and bone marrow) serve as *Leishmania* host cells [97,98]. Approaches in human medicine to further enhance the efficacy of intracellular uptake include liposomes with macrophage-specific ligands (e.g., mannose, tuftsin residues, phosphatidylserine) [78]. In dogs, meglumine antimonate had a longer (plasma) half-life when it was encapsulated in liposomes, which could be of benefit for a 24 h administration interval [99]. Intravenous injections of low doses of meglumine antimoniate encapsulated in liposomes in 14 dogs infected with *L. chagasi* were proven to promote higher antimony concentrations in bone marrow (2-fold), liver (63-fold), and spleen (68-fold), compared to higher injected doses of meglumine antimoniate (non-liposomal) in five dogs [100]. However, even with liposomal meglumine antimoniate, complete *Leishmania* clearance was not achieved. In a study on nine dogs naturally infected with *L. chagasi* in Brazil, cultures of bone marrow samples were positive in all dogs 120 days after treatment, although parasites were not detected in bone marrow smears 30 days after termination of treatment [101]. In another Brazilian study on the treatment with liposome encapsulated meglumine antimoniate in twelve dogs, immunocytochemical investigation revealed negative results in livers and lymph nodes 150 days after treatment. However, bone marrow cultures were positive in all dogs. The parasite burden of the lymph node, liver, and spleen was significantly lower compared to groups of dogs that received either empty liposomes (*n* = 12) or saline (*n* = 12). Although no significant reduction of *Leishmania* load was observed in skin samples, xenodiagnostic methods revealed a significantly lower infectivity to sandflies in dogs treated with liposomal meglumine antimoniate (0.65% infection efficacy) compared to dogs given empty liposomes (14.3% infection efficacy) and dogs that received saline (21.5% infection efficacy). However, xenodiagnostic studies were not conducted in dogs before treatment and there was no group of control dogs receiving meglumine antimoniate without encapsulation [102]. Treatment outcomes with liposomal meglumine antimoniate also can be improved by adding allopurinol. Combined treatment led to the best clinical outcome and was mostly effective in reducing the parasite burden of the bone marrow and spleen (*n* = 6) when compared to groups of dogs treated with (1) liposomal meglumine antimoniate, (2) allopurinol and saline, (3) allopurinol and empty liposomes, (4) empty liposomes, and (5) saline. Parasitological cure (negative culture of bone marrow and negative PCR of bone marrow, spleen, liver, and skin and no sandfly infectivity) was reached by half of the dogs (*n* = 3) 200 days after treatment [103]. A further study dealt with a modified liposome formulation (PEGylated), which is supposed to circulate in blood for longer times and was shown to have a better effect on skin parasite load (significant reduction) than the non-modified liposome formulation [104]. However, the use of liposomes in general carries the risk of different adverse effects, among which tachypnoea, sialorrhea, defecation, and lethargy are most common; they typically occur within the first 15 min after application due to an activation of the complement system [102]. Furthermore, there is no commercially liposomal formulation of meglumine antimoniate available and thus, its use is limited to scientific studies for the moment.

#### 3.1.2. Adverse Effects

In dogs, meglumine antimoniate is mainly eliminated (>80%) through the kidneys by glomerular filtration within 9 h after application [84]. A main limiting factor in the treatment of dogs is its nephrotoxic effect, which can lead to acute renal failure [88,105]. Tubular damage (cell swelling, necrosis, and apoptosis) due to meglumine antimoniate treatment (100 mg/kg, q24h, SC, for 28 days) was shown in four out of four uninfected, healthy dogs of which renal biopsy specimens were taken before and 28 days after the end of treatment; none of the dogs showed any clinical signs of impaired kidney function before, during, and after treatment. In contrast, no abnormal findings were detected in the glomerula of the four dogs. Urinalysis performed at the end of the 28-day treatment period revealed an increased protein level in two out of four dogs [106]. In infected dogs, it is discussed whether treatment with meglumine antimoniate might cause glomerular lesions indirectly by parasite death-induced formation and deposition of circulating immune complexes [105]. However, there is a lack of histopathological studies on infected dogs. Clinical trials on the course of parameters indicating glomerular and tubular function were mainly performed on dogs that did not receive meglumine antimoniate monotherapy, but only in combination with allopurinol; remission of proteinuria and/or a significant decrease in UP/C in some dogs, as well as deterioration or no changes at all, were observed [107,108,109]. Kidney function deteriorated during combined treatment in one out of twelve naturally infected dogs in a study in Spain, in which overall no significant changes were observed in the glomerular filtration rate and urine specific gravity (USG) after treatment [105]. Overall, thorough evaluation of kidney function before and monitoring during and after treatment with meglumine antimoniate is considered essential.

Since a temporary increase in liver enzyme activities (alanine aminotransferase (ALT), aspartate aminotransferase (AST), alkaline phosphatase (AP)) during treatment was observed in a study, hepatotoxic effects might also be related to meglumine antimoniate treatment [110]. If meglumine antimoniate is administered subcutaneously or intramuscularly, local reactions on the injection site (pain and swelling) can occur, whereas intravenous injections might cause thrombophlebitis [86,111].

In a study conducted in Italy, adverse effects of combined treatment with meglumine antimoniate (50 mg/kg, q12h, SC, for 30 days) and allopurinol (10 mg/kg, q12h, PO, for 6 months) were recorded retrospectively in 26/87 dogs and included injection-related local (swelling, granuloma, abscess) or systemic reactions (lethargy, anorexia, immobility) (*n* = 10), gastrointestinal signs (diarrhea and vomiting) (*n* = 8), impaired kidney function, acute pancreatitis (*n* = 5) and (severe) skin reactions (*n* = 3). In 13/26 dogs, treatment was interrupted due to adverse effects. In a prospective evaluation of 16 dogs, severe local reactions at the injection site occurred in 2/16 dogs. Furthermore, self-limiting subcutaneous reactions (*n* = 2), diarrhea (*n* = 1), and increased liver enzyme activity (*n* = 1) were observed [112]. Cases of acute pancreatitis during meglumine antimoniate treatment have also been described in other studies [42,112,113]. In a clinical trial on 20 dogs treated with meglumine antimoniate, 4/20 dogs had increased canine pancreatic lipase values, 3/20 dogs had clinical signs compatible with pancreatitis and 2/3 dogs ultrasonographic findings indicative for pancreatitis [114]. In a multicenter study conducted in Spain, 14/33 dogs were diagnosed with clinical pancreatitis during or at the end of treatment with meglumine antimoniate (100 mg/kg, q24h, SC, for 30 days) in combination with allopurinol (10 mg/kg, q12h, PO, for 1 year) and, occasionally, prednisone (0.7 mg/kg, q24h, PO, for 7–10 days) [115]. In contrast, in a controlled prospective study, in which 20 dogs with leishmaniosis were treated with meglumine antimoniate (100 mg/kg, q24h, SC) and allopurinol (10 mg/kg, q12h) for 28 days, there was no evidence (neither by clinical nor by laboratory diagnostics performed during and at the end of treatment) of pancreatitis. No cardiac adverse effects (increased serum cardiac troponin I (cTnI) or clinical evidence), which often occur in human medicine, were observed in the dogs [116]. Cardiotoxic effects were also not reported in another study on 28 naturally infected dogs, since findings in cTnI and electrocardiography were normal before and after a 60-day treatment period with meglumine antimoniate (75 mg/kg, q12h, SC) [117].

#### 3.1.3. Drug Resistance Potential

Since meglumine antimoniate is used for treatment in human and veterinary medicine, the risk of development and transmission of resistant *Leishmania* strains is a special one-health concern. However, meglumine antimoniate is used commonly and not prohibited in veterinary medicine. To ensure treatment success in humans, the WHO endorses choosing only treatment options for dogs that are not applied in human medicine [75]. In an in vitro study on *Leishmania* strains isolated from three humans (before and after treatment) and two dogs (before and during allopurinol treatment) from Portugal, the lowest meglumine antimoniate susceptibility (highest half-maximum inhibitory concentrations) was proven for the isolates (promastigote stages) of the two dogs, although they did not receive meglumine antimoniate before. Frequent use of this drug in the Portuguese dog population might be responsible for the low susceptibility [76]. Indeed, high drug susceptibility was proven in a study on pentavalent antimony susceptibility of intracellular *Leishmania* stages isolated from 24 privately owned dogs in an endemic area in Algeria without drug pressure [118]. In particular, repeated treatment cycles, performed in case of disease relapse, are thought to carry the risk of reduced treatment response due to selection of resistant *Leishmania* strains. In a study (endemic area without drug pressure) including three dogs experimentally infected with *L. infantum*, pentavalent antimonial susceptibility in vitro was highest in strains isolated from the dogs before treatment, followed by the ones isolated after treatment with meglumine antimoniate once, and finally the ones obtained after disease relapse and a second treatment cycle with meglumine antimoniate in a liposomal formulation [119]. In another in vitro study from Spain, two different *Leishmania* strains were isolated from a dog with leishmaniosis before and after treatment with two courses (1 month apart) of meglumine antimoniate. The dog’s clinical score increased after the first treatment cycle and returned to pre-treatment levels after the second. In the follow-up period, clinical signs worsened again. Since a significant increase in the isolates’ IC50 was recorded after the treatment cycles, the authors concluded that disease relapse occurred due to drug resistance [120]. In contrast, in an in vivo study (mouse model), in which decreased drug susceptibility was also observed after meglumine antimoniate treatment of four dogs, the number of treatment cycles was not decisive for development of resistance; isolates from a dog treated with meglumine antimoniate six times had a resistance index comparable with that of isolates from two dogs treated with meglumine antimoniate three times [121]. Studies with higher numbers of dogs are needed to draw valid conclusions on the development of meglumine antimoniate resistance mechanisms in *Leishmania*.

### 3.2. Miltefosine

A more recent leishmanicidal treatment option is the hexadecylphosphocholine miltefosine. Miltefosine was originally used as anticancer treatment in human medicine and is an analogue of the cell membrane component phosphatidylcholine (lecithin) [122,123,124,125]. The toxic effect on *Leishmania* is based on a disruption of the parasitic phospholipid metabolism, Ca^2+^ homeostasis, and inhibition of cytochrome c oxidase, which are considered essential for the intracellular survival of the parasites (Figure 1) [126]. Furthermore, it is assumed that miltefosine affects the parasites indirectly by increasing the host’s T-cell and macrophage activity and the production of reactive oxygen and nitrogen intermediates with microbiocidal effect [127,128,129]. Miltefosine is available as a suspension for oral application, which should be given together with food. A long terminal elimination half-life in dogs of approximately 160 h and resulting drug accumulation (factor of 7.65 ± 1.99 during 28-days of treatment) cause a prolongation of the therapeutic effect and improvement of signs for at least 1 month beyond the application period, during which, however, clinical improvement can already be observed [124,130].

#### 3.2.1. Initial Treatment

For miltefosine monotherapy, studies with experimentally infected dogs and with dogs living in non-endemic areas are lacking. In different clinical trials on dogs with naturally acquired leishmaniosis in endemic areas, it was shown that miltefosine monotherapy leads to an improvement of disease signs (Table 2 and Table 5). In a controlled, randomized multicenter study, miltefosine treatment (2 mg/kg, q24h, PO, for 28 days) of 60 naturally infected dogs led to a significant clinical improvement (reduction of clinical scores by 51%) and antiparasitic efficacy (90% of dogs with negative bone marrow cytology) until the end of the 6-week observation period; a decrease in antibody titers (IFAT) by treatment was recorded in 9% of the dogs [88]. In another multicentric clinical trial on naturally infected dogs, efficacy (*n* = 82) and safety (*n* = 94) of miltefosine treatment (2 mg/kg, q24h, PO, for 28 days) were investigated. Clinical improvement (decline in clinical scores) with a significant time-dependent effect was observed during the 2-month observation period. Complete remission of clinical signs was reached in 20% of the dogs. An improvement was also observed in hematological and biochemical alterations. Approximately 50% of the dogs which initially had hematological alterations (anemia in 36%, thrombocytopenia in 56%, and leukocytosis in 43%) had none at the end of the observation period. A significant drop was observed in the percentage of dogs with hypalbuminemia (83% to 23%) and low a/g ratios (76% to 25%). *Leishmania* parasites were no longer detectable in bone marrow smears of 17 (out of 33) dogs at the end of the observation period. *Leishmania* antibody titers (IFAT) declined in 37% of the dogs with a 2-fold decrease [124]. Clinical improvement, as well as a reduction in parasite burden (qPCR of skin biopsies) and sandfly infectivity (xenodiagnoses) following miltefosine monotherapy (2 mg/kg, q24h, PO, for 28 days), was observed during treatment and a subsequent 2-month follow-up period in a study conducted on 35 dogs in Brazil. A significant reduction in clinical scores, with clinical improvement in 94% of the dogs was recorded. Until the end of the observation period, skin *Leishmania* loads (qPCR) were reduced by 99%; a total of 74% of the dogs were not infectious to sandflies (anymore) [131]. However, as is the case with every other treatment option, monotherapy with miltefosine might not lead to parasitological cure and a re-increase in parasite burden (after initial decrease) can occur. In a study conducted in Italy on 18 dogs with leishmaniosis, the effect of miltefosine treatment (2 mg/kg, q24h, PO, for 30 days) on parasite loads in the blood and lymph nodes was investigated during a 1-year observation period. After 30 days of treatment, an improvement of clinical signs was observed in 90% of the dogs. *Leishmania* DNA load in lymph nodes was highest at the time of diagnosis and decreased after treatment in all samples, mostly between 5 and 6 months. Parasite detection in the blood was irregularly possible throughout the observation period with a marked decrease in parasite load from month 2–4. After 9–12 months, a re-increase was observed in the parasite load of lymph nodes and blood in some dogs and accompanied by disease relapse [132]. However, since this study was conducted in an endemic area, it remains unclear whether the increases in parasite loads are attributable to insufficient parasite elimination by miltefosine or to re-infection. In a study conducted in Brazil, *Leishmania*-infected dogs without (1/14 dogs), with few (5/14 dogs), and with multiple (8/14 dogs) signs of the disease were treated with different miltefosine dosing regimens, which, however, all differed from the commonly recommended dosage. Miltefosine was applied at 100 mg/dog/day for 28 days to 5/14 dogs, at 200 mg/dog/day for 28 days to 5/14 dogs, and at 100 mg/dog/day for 45 days to 4/14 dogs. Unfortunately, information on the bodyweight of the included dogs is not given, which is why the dosages cannot be converted to mg/kg. The treatment led to a significant improvement of clinical signs; at the end of the 2-year observation period, 7/14 dogs had no, 3/14 dogs had a few, and 2/14 dogs had multiple signs of the disease, whereas 2/14 dogs died (after 22 and 23 months) before observation period ended. *Leishmania* IgG levels (ELISA) were significantly lower on day 180 than before treatment onset and at the end of the observation period. Interferon gamma (IFN-γ) trended to increase by treatment, although not statistically significant in any group. Interleukin (IL)-4 decreased significantly within 180 days after treatment with 100 mg miltefosine/dog/day given for 28 or 45 days, whereas no significant changes were observed in levels of IL-10 (trend to decrease until day 180 and re-increase until day 300 in all groups). Despite treatment, *Leishmania* DNA was detected by qPCR in bone marrow samples during the entire observation period. Bone marrow parasite loads (determination possible in 9/14 dogs) decreased significantly in the majority (66%) of dogs within 3 months, whereas a progressive increase was recorded in the remaining dogs following treatment (irrespective of dosing regimen). After 6 months, parasite loads increased significantly in all dogs. After euthanasia of the dogs, *Leishmania* DNA was detected in spleen samples of 13/14 dogs. Due to inadequate parasite clearance, the authors concluded that treatment with miltefosine, irrespective of the dose, should not be performed in endemic areas [133]. However, since the aforementioned studies were conducted in endemic areas, re-infection during observation period cannot be excluded.

A synergistic effect of combined treatment with miltefosine and allopurinol, as shown for meglumine antimoniate and allopurinol, was investigated in several studies with different treatment groups (Table 2 and Table 5). In a study on 45 naturally infected dogs in Brazil, groups of dogs (*n* = 15) were compared, treated either with miltefosine (2 mg/kg, q24h, PO, for 28 days) combined with allopurinol (20 mg/kg, q12h, PO, for 28 days), miltefosine monotherapy (2 mg/kg, q24h, PO, for 28 days) or allopurinol monotherapy (20 mg/kg, q12h, PO, for 28 days). At the end of the 28-day treatment period, significant clinical improvement (decrease in clinical score) was observed in all groups, with 52.6% of reduced clinical scores in dogs that received combined treatment, 36.9% in dogs with miltefosine monotherapy, and 58.4% in dogs with allopurinol monotherapy. Direct detection of *Leishmania* by qPCR of skin samples was possible in all dogs before and after treatment. The parasite loads decreased significantly in dogs treated with miltefosine combined with allopurinol and in dogs with allopurinol monotherapy and correlated with the clinical score but not with *Leishmania* antibody titers (IFAT), which did not change significantly in the short observation period [57]. In another study conducted in Brazil, the same three treatment protocols were compared (allopurinol group from the aforementioned study) with special regard to an effect on laboratory alterations. Dogs were treated either with miltefosine (2 mg/kg, q24h, PO, for 28 days; *n* = 15), allopurinol (20 mg/kg, q12h, PO, for 28 days; *n* = 15), or combined treatment (dosages as in the monotherapy groups; *n* = 15). After 28 days of treatment, a significant reduction in clinical scores in all groups and an increase in red blood cells, significant in the combined treatment and allopurinol group, were observed. In dogs with combined treatment, a/g ratios increased significantly. Initially increased total protein, globulin, and antibody titers persisted high, while levels of serum creatinine, urea, and activity of ALT remained within the reference range in all groups. No significant differences were observed in the levels of cytokines (IL-2, IL-6, IL-10 and IFN-y) between the groups [56]. The short-term effects of miltefosine (2 mg/kg, q24h, PO, for 28 days) and allopurinol (20 mg/kg, q12h, PO) were compared to miltefosine monotherapy in another study conducted in Brazil on 30 naturally infected dogs. Clinical signs improved (significant reduction in clinical scores) in both groups until the end of treatment but there were no significant changes in laboratory alterations. In dogs that received combined treatment, significant increase in albumin (which, however, was below the reference range before and after treatment) was observed. The number of dogs with proteinuria increased in the miltefosine monotherapy group from 11/15 to 12/15, whereas it decreased from 13/15 to 11/15 in the combined treatment group. In UP/C ratios, no significant differences were observed before and after treatment. Among the urinary parameters used to monitor renal impairment (cystatin C and lipocalin-2 indicating tubular damage, microalbumin indicating glomerular damage), significant reduction was only observed in lipocalin-2 levels of dogs treated with miltefosine and allopurinol [134]. In a controlled study, combined treatment with miltefosine (2 mg/kg, q24h, 4 weeks) and allopurinol (10 mg/kg, q12h, PO, for at least 6 months) led to a decrease in initially increased AST/ALT activity and a significant decrease in initially increased urea-nitrogen levels (restoration to normal). Furthermore, altered urinary parameters were normalized by treatment. Alpha-2 globulin levels normalized within 3 months. Although total protein and gamma-globulin levels remained high, all dogs turned antibody-negative (IFAT) until the end of the 6-month observation period. Parasites were not detected in the lymph node and bone marrow smears of any dog 3 months after treatment initiation. Complete remission of clinical signs was observed 3 months after treatment initiation in all dogs. For combined treatment with miltefosine and allopurinol, a trend to normalized cytokine gene expression was found as well [92]. A significant improvement of clinical signs was also observed in another controlled study on eight dogs treated with miltefosine (2 mg/kg, q24h, PO, for 28 days) in combination with allopurinol (10 mg/kg, q12h, PO, for 6 months). Significant changes in acute phase protein levels were only observed for HP (within 3 months after treatment initiation) and not for CRP, ferritin, albumin, and PON-1 activity [93]. In a multicentric controlled study, treatment with miltefosine (2 mg/kg, q24h, PO, for 28 days) in combination with allopurinol (10 mg/kg, q12h, PO, for 7 months) was applied to 37 dogs. Within 3 months from treatment onset, clinical signs improved significantly; until the end of the observation period, mean clinical score was reduced by 89.9%. Albumin/globulin ratios increased by treatment and antibody titers (IFAT) decreased significantly within the first month and between the first and third month. A significant reduction was observed in the bone marrow parasitic load (PCR) within 1 month from treatment onset [91]. In a study conducted in Italy, the efficacy of a combined treatment with miltefosine (2 mg/kg/day, PO) and allopurinol (10 mg/kg/day, PO) for 30 days, followed by allopurinol monotherapy for 11 months, was evaluated in 28 dogs with leishmaniosis during a 1-year observation period. Significant clinical improvement, a decrease in *Leishmania* antibody titers (IFAT) and a reduction of the parasite load (PCR) in lymph nodes but not in blood was observed in 24/28 dogs which completed the 1-month treatment period. Within the first 6 months, miltefosine treatment was repeated in 8/24 dogs; in 4/8 dogs due to the re-emergence of clinical signs (three dogs in the third month and one dog in the fourth month after treatment initiation) and in 4/8 dogs to investigate the effect of repeated miltefosine cycles, especially on parasite clearance. Repeated treatment cycles (regardless of the reason) did not lead to complete parasitological cure, but in re-treated dogs without clinical relapse, *Leishmania* load of lymph nodes decreased significantly [135]. A multicenter study conducted in Brazil aimed to evaluate the efficacy of miltefosine treatment on 21 naturally infected dogs. Miltefosine treatment (2 mg/kg, q24h, PO, for 28 days) in some cases was combined with/followed by allopurinol (10–15 mg/kg, q12h, PO, for 30–180 days; *n* = 18) and/or domperidone (0.5–1.1 mg/kg, q12h, PO, for 30–180 days; *n* = 16), vaccination (*n* = 3), omega-3 fatty acids (*n* = 3), or marbofloxacin (*n* = 1). From treatment onset, a progressive improvement of clinical signs was observed in the dogs. However, in 3/21 dogs, clinical scores increased between the end of miltefosine treatment and the 6-month observation period (but not above pre-treatment levels). Overall, there was a reduction in the number of dogs assigned to more severe disease stages (considering clinical and laboratory parameters) and an increase in dogs mildly affected by the disease. Histopathological examination of skin samples indicated a decrease in inflammatory response (infiltrate pattern) and macrophages containing amastigotes after miltefosine treatment. Furthermore, a significant decrease was observed in the skin parasite load (PCR) of all dogs within 1 month and was maintained in 18/21 dogs until the end of the follow-up period. After 6 months, parasites were not detectable in the skin of 8/21 dogs, whereas 3/21 dogs (all treated with miltefosine only) showed an increased load, indicating disease relapse [136]. In another study conducted in Italy, two different dosing regimens of miltefosine in combination with allopurinol were investigated. In total, 18/34 dogs were treated with miltefosine standard doses (2 mg/kg, q24h, PO) for 28 days and 16/34 dogs were treated with an altered treatment regimen including lower miltefosine dose (1.2 mg/kg, q24, PO) during the first 5 days, followed by a higher dose (2.5 mg/kg, q24, PO) for the following 25 days. An improvement in clinical signs was observed within 2 months after treatment initiation, similar in both groups. Significant differences between both groups were not observed in UP/C ratios, *Leishmania* antibody titers (IFAT), or in parameters indicating kidney and liver function, whereas after 3 and 6 months, groups differed significantly in hematocrit, hemoglobin and red blood cells, albumin, globulin, and a/g ratio (all higher in altered miltefosine regimens). Parasite detection after 2 months was possible in the lymph node smears from one dog of each group and in PCR of bone marrow in 12/14 dogs with regular, and 7/14 dogs with altered miltefosine treatment; the decrease in parasite load was not significant. Disease relapse, including the worsening of clinical signs, a decrease in hematocrit, and a decrease in body weight, occurred in four dogs with a standard miltefosine dose (1/4 after 90 days, 3/4 after 180 days); parasites were visualizable in lymph node smears of all four dogs. Since the altered miltefosine dosing regimen did not promote relapses and was associated with good clinical/parasitological efficacy, further studies are needed to evaluate this miltefosine regimen in a higher number of dogs [137].

The long-term effect of miltefosine treatment was retrospectively evaluated in nine dogs that received miltefosine (2 mg/kg, q24h, PO, for 30 days) and allopurinol (10 mg/kg, q24h, PO, for 6 years). A significant improvement of clinical score was observed within 3 months. *Leishmania* load, quantified by PCR of lymph node samples, decreased significantly within 3 months (35-fold). During the observation period, disease relapses were observed in four out of nine dogs; disease relapses were observed in two out of four dogs after 6 months, in the remaining two of the four dogs, after 28 and 48 months. *Leishmania* antibodies (IFAT) decreased with treatment, but significant differences compared to baseline titers were recorded only from month 9 on [70]. In another retrospective study, clinical records of 173 dogs with leishmaniosis from an endemic area were reviewed to obtain information about the long-term (3.2–9 years) effect of combined treatment with miltefosine (2 mg/kg, q24h, PO, for 28 days) and allopurinol (10 mg/kg, q12h, PO, for 2–12 months). Disease relapse and the repetition of miltefosine treatment was recorded for 30 dogs after a mean time of 27.2 months after the first treatment cycle. A third treatment cycle was necessary in two dogs after a mean time of 16.9 months from the second treatment cycle, and one dog received five treatment cycles. After treatment initiation, clinical improvement (significant for all signs) was observed in 170/173 dogs (98%) during the follow-up in a mean time of 3 months. After a mean time of 16.7 months, 152/173 dogs (88%) dogs achieved complete remission of clinical signs. In 29/30 dogs with disease relapse, which were re-treated, clinical signs improved in the follow-up period; the mean time was 8.6 months. Improvement of clinicopathological alterations (significant for all signs) was observed in 171/173 dogs after a mean time of 4.1 months from treatment initiation and in 28/30 re-treated dogs with relapses after a mean time of 11 months. A decrease in antibody levels (ELISA) was recorded after a mean time of 2.6 months from treatment initiation and 7.8 months from a second treatment cycle. At some timepoint (mean time 10.5 months) after treatment initiation, 158/173 dogs were antibody-negative and 24/30 dogs (mean time 15.6 months) were antibody-negative after a second treatment cycle [138]. Due to the overall good efficacy of miltefosine in dogs with leishmaniosis, combined treatment with miltefosine and allopurinol is considered a valuable alternative first-line treatment option [2].

Recent attempts to reach higher antiparasitic efficacy consists of the use of the miltefosine derivative oleylphosphocholine (OlPC) in the treatment of canine leishmaniosis [139]. Although there is only minor chemical difference to miltefosine, preliminary clinical data indicate a beneficial outcome of treated dogs. In a controlled, randomized, and observer-blinded clinical study on naturally infected dogs in Brazil, treatment with OlPC (4 mg/kg, q24h, PO, for 28 days; *n* = 17) was compared to miltefosine (2 mg/kg, q24h, PO, for 28 days; *n* = 16). Clinical signs of the dogs treated with OlPC improved; mean clinical scores were significantly lower than scores from miltefosine-treated dogs after 3 months and this difference persisted until the end of the 6-month observation period. The hematocrit of the (initially anemic) dogs treated with OlPC increased but remained below the reference range after 6 months. A decrease was observed in globulin levels for OlPC, but not for miltefosine-treated dogs. In contrast, creatinine levels of the OlPC group showed an increase (to levels slightly above the reference range) within the observation period, whereas no changes were observed in miltefosine treated dogs. Parasite loads in spleen aspirates were significantly reduced within 6 months only in dogs treated with OlPC. Overall, these first results are promising, but further studies, especially on adverse effect potential and long-term outcome are needed [140].

**Table 5:** Treatment of *Leishmania*-infected dogs with miltefosine alone and combined with allopurinol and comparison to treatment with meglumine antimoniate alone and meglumine antimoniate combined with allopurinol in the last 25 years.

Design	Dosage	Length		Dogs		Control Intervals	Outcome				Adverse Effects	Ref.
		T	O	Incl.	Excl.		Clinical Signs	Laboratory Alterations	Parasite Detection			
									Indirect	Direct		
uncontrolled	**MI** 2 mg/kg q24h	30 d	1 y	18	--	every month	improvement in 90% of dogs within 1 m, relapse in some dogs after 9–12 m	n.a.	decrease in titer within 1 m	decrease in load (PCR) in PB and LN (mostly between 2–4 m and 5–6 m), re-increase in some dogs with relapse after 9–12 m	n.a.	[132]
uncontrolled	**MI** 2 mg/kg q24h	28 d	56 d	82 efficacy 94 safety	--	0, 14, 28, 42, 56 d	score reduction * of 61%, remission in 20% of dogs	normalization of hematological alterations in 50% of dogs but without * difference in number of dogs with specific alterations, less * dogs with low alb and a/g	decrease in IFAT titer (2-fold in 37%, 4-fold in 4% of dogs)	neg. BM cytology in 17/33 initially pos. dogs	mainly GI	[124]
controlled not randomized	**MI** 100 mg/**dog** /day	28 d	2 y (euth.)	5	overall 2 (death after 22, 23 m)	0 d, after T, every 3 m	improvement *, remission in 7/14 dogs after 2 y	persistent abnormal urea, increase in bili in group 3 after T, decrease * of IL-4 in groups 1 and 3, no * change in IL-10 and IFN-γ	decrease * of IgG (ELISA) after 180 d	decrease * in BM load (PCR) in 66% of dogs within 3 m, (re-)increase * in all dogs after 6 m, pos. spleen PCR in 13/14 dogs after 2 y (euth.)	vomiting	[133]
	**MI** 200 mg/**dog** /day	28 d	2 y (euth.)	5								
	**MI** 100 mg/**dog** /day	45 d	2 y (euth.)	4								
uncontrolled	**MI** 2 mg/kg q24h	28 d	3 m	35	--	0, 2, 4, 6, 8, 10, 12 w	weight gain *, score reduction * of 68%, improvement in 94% of dogs	n.a.	n.a.	decrease * in load (PCR) in skin (99%) and sandfly infectivity (18/35 to 9/35 dogs)	none	[131]
controlled randomized observer-blinded	**MI** 2 mg/kg q24h	28 d	6 m	16	--	0, 1, 2, 3, 4, 5, 6 m	no score reduction	persistent increased glob, no change in crea	n.a.	no * decrease in load (PCR) in spleen	n.a.	[140]
controlled randomized	**MI** 2 mg/kg q24h	28 d	6 w	60	overall 29 MI+MA	every 14 d	score reduction * of 51%, no * intergroup difference	steady cbc and biochemistry w/o * intergroup difference in course, lower a/g *, higher tp * than MA dogs, increased γ-GT in 2% of dogs	decrease in IFAT titer in 9% of dogs, no correlation with clin. score	neg. BM cytology in 90%, no * intergroup difference	GI, general condition, PU/PD	[88]
	**MA** 100 mg/kg q24h or split q12h SC	28 d	6 w	25 34	(lost to follow-up)	every 14 d	score reduction * of 63%, no * intergroup difference	steady cbc and biochemistry w/o * intergroup difference in course, higher a/g * and lower tp * than MI dogs, increased γ-GT in 20% and crea in 11% of dogs	decrease in IFAT titer in 33% of dogs; no correlation with clin. score	neg. BM cytology in 91%, no * intergroup difference	GI, general condition, others (e.g., injection- site)	
controlled not randomized	**MI** 2 mg/kg q24h	28 d	29 d	15	--	0, 29 d	score reduction * of 3.5 points, no * difference to MI+AL	no * reduction of score, more dogs with proteinuria (11/15 to 12/15 dogs), no * difference in UP/C and USG, no * change of urinary NGAL, CisC and microalb	n.a.	n.a.	none	[134]
	**MI** 2 mg/kg q24h +**AL** 20 mg/kg q12h	28 d	29 d	15	--	0, 29 d	score reduction * of 6.1 points, no * difference to MI	no * reduction of score, increase * of alb, less dogs with proteinuria (13/15 to 11/15), no * difference in UP/C and USG, decrease * of NGAL, no * change in CisC and microalb	n.a.	n.a.	none	
uncontrolled	**MI** 2 mg/kg q24h +**AL** 10 mg/kg /day	30 d 12 m	12 m	28	4 (adv. effects, death)	0, 1, 3, 6, 9, 12 m	improvement * within 1 m, re-treatment in 8/24 dogs (each 4 dogs with and w/o relapse) within 6 m	n.a.	decrease in IFAT titer within 1 m	decrease * of load (PCR) in LN within 1 m; after re-treatment of dogs w/o relapse decrease * in LN	GI, reduced rbc and wbc	[135]
controlled randomized	**MI** 2 mg/kg q24h +**AL** 10 mg/kg q12h	28 d 180 d	180 d	18	3 (death, relapse)	0, 30, 60, 90, 180 d	score reduction of 62% within 2 m, no * intergroup difference, relapse in 4 dogs between 90 and 180 d	lower * hct, hb, rbc, alb, glob, a/g than other group after 90 and 180 d	decrease or persistence of IFATtiter	pos. BM PCR in 12/14 dogs after 2 m, LN cytology in 1 dog, no * decrease in load in BM within 60 d, pos. LN cytology in 4/4 relapsed dogs	GI	[137]
	**MI** 1.2 mg/kg q24h, then 2.5 mg/kg q24h +**AL** 10 mg/kg q12h	5 d 25 d 180 d	180 d	16	--	0, 30, 60, 90, 180 d	score reduction of 72% within 2 m, no * intergroup difference	higher * hct, hb, rbc, alb, glob, a/g than other group after 90 and 180 d	decrease or persistence of IFAT titer	pos. BM (PCR) in 7/14 dogs after 2 m, LN cytology in 1 dog, no * decrease in load in BM within 60 d	GI	
retrospective	**MI** 2 mg/kg q24h +**AL** 10 mg/kg q12h	28 d 2–12 m	3.2–9 y	173	--	12 irreg. timepoints	improvement * (after 3 m) in 170, remission (16.7 m) in 152, relapse (27.2 m) in 30/173 dogs, improvement in 29/30 re-treated dogs (after 8.6 m), repeated relapse in 3/30 dogs	improvement (each alteration *) in 171/173 dogs (after mean 4.1 m) and 28/30 relapsed dogs (after mean 11 m)	decrease in ELISA level (after 2.6 m; 7.8 m after re-treatment), neg. at some point in 158 dogs (24/30 re- treated dogs)	n.a.	nausea/ vomiting	[138]
uncontrolled	**MI** 2 mg/kg q24h +/− **AL** 10–15 mg/kg q12h +/-**immuno**- **therapy**	28 d 30–180 d	180 d	21 (3/21 w/o signs)	--	0, 30, 180 d	progressive score reduction, increase in 3 dogs after T, less dogs in severe disease stages	decrease in inflammatory skin response, less dogs in severe disease stages	n.a.	decrease * in load (PCR) in skin in all dogs within 1 m, re-increase in 3/21 dogs after 6 m, neg. skin PCR in 8/21 dogs	n.a.	[136]
controlled randomized	**MI** 2 mg/kg q24h +**AL** 10 mg/kg q12h	28 d 7 m	7 m	37	3 (lost to follow-up)	0, 14, 28, 84, 140, 196 d	improvement * within 3 m, score reduction of 90%, no * intergroup difference	increase in a/g within 3 m, no * intergroup difference in course of a/g, γ-glob, UP/C	decrease * in IFAT titer within 1 and 3 m, no * intergroup difference	decrease * in load (PCR) in BM within 1 m, intergroup difference * after 84 d (higher in MI)	none	[91]
	MA 50 mg/kg q12h, SC +**AL** 10 mg/kg q12h	28 d 7 m	7 m	36	4 (lost to follow-up)	0, 14, 28, 84, 140, 196 d	improvement * within 3 m, score reduction of 84%, no * intergroup difference	increase in a/g within 3 m, no * intergroup difference in course of a/g, γ-glob, UP/C	decrease * in IFAT titer after 3 m, no* intergroup difference	decrease * in load (PCR) in BM within 1 m, intergroup difference * after 84 d (higher in MI)	vomiting, asthenia	
retrospective	**MI** 2 mg/kg q24h +**AL** 10 mg/kg q24h	30 d 6 y	6 y	9	--	0, 1 m, every 3 m for 2 y, every 12 m	improvement * within 3 m, remission in 6/9 dogs after 1 y, relapse in 4/9 dogs (after 6, 28 and 48 m)		decrease * in IFAT score after 9 m, increase in dogs with relapse	decrease * in load (PCR) in LN within 3 m (35-fold), increase in dogs with relapse	itching (AL)	[70]
	**MA** 100 mg/kg q24h SC +**AL** 10 mg/kg q24h	30 d 6 y	6 y	9	--	0, 1 m, every 3 m for 2 y, every 12 m	improvement * within 1 m, relapse in 1/9 dogs after 1 y remission in 9/9 dogs after 15 m		decrease * in IFAT score after 1 m, increase in dogs with relapse	decrease * in load (PCR) in LN within 1 m (50-fold lower after 3 m) until 9 m, increase in dog with relapse	itching (AL)	
controlled not randomized	**MI** 2 mg/kg q24h +**AL** 10 mg/kg q12h	28 d 6 m	3 m	10	2 (adv. effects)	0, 30, 90 d	score reduction * within 1 and 3 m, no * intergroup difference	decrease * of HP after 3 m, abnormal CRP in 2/8 dogs, HP in 5/8 dogs, ferr in 7/8 dogs, PON-1 in 1/8 dogs, alb in 3/8 dogs	n.a.	n.a.	vomiting	[93]
	**MA** 50 mg/kg q12h SC +**AL** 10 mg/kg q12h	28 d 6 m	3 m	10 + 2 excl. MI	--	0, 30, 90 d	score reduction * (all timepoints), no * intergroup difference	difference * in CRP, ferr, PON-1, alb; abnormal CRP in 2/12 dogs, HP in 9/14 dogs, ferr in 9/12 dogs, PON-1 in 0/12 dogs, alb in 3/12 dogs	n.a.	n.a.	acute renal failure	
controlled not randomized	**MI** 2 mg/kg q24h +**AL** 10 mg/kg q12h	4 w 6 m at least	6 m	6 at risk of hepatic/renal failure	--	0, 1, 2, 3, 6 m	remission within 3 m	decrease * and restoration of urea, decrease in ALT/AST, persistent high tp and γ-glob, normalization of α-2-glob within 3 m and urinalysis results, trend to normalization of cytokine gene expression	neg. IFAT in 5/6 dogs after 3 m, in 6/6 dogs after 6 m	neg. LN and BM cytology after 3 m	n.a.	[92]
	**MA** 100 mg/kg q24h (route not given) +**AL** 10 mg/kg q12h	4 w 6 m at least	6 m	6	--	0, 1, 2, 3, 6 m	remission within 3 m	normalization ALT/AST, a/g within 2 m, tp and γ-glob within 3 m, trend to normalization of cytokine gene expression	neg. IFAT in 6/6 dogs after 3 m	neg. LN and BM cytology after 3 m	n.a.	

AL, allopurinol administered orally; adv., adverse effects; alb, albumin; ALT, alanine aminotransferase; AST, aspartate aminotransferase; a/g, albumin/globulin ratio; BM, bone marrow; cbc, complete blood count; CisC, cystatin C; crea, creatinine; CRP, C-reactive protein; d, day; ELISA, enzyme-linked immunosorbent assay; euth, euthanasia; Excl.; excluded during observation period; ferr, ferritin; GI, gastrointestinal; glob, globulin; hb, hemoglobin; hct, hematocrit; HP, haptoglobin; IFAT, immunofluorescence antibody test; IFN, interferon; IgG, immunoglobulin G; IL, interleukin; Incl., included for treatment; kg, kilograms; LN, lymph node; m, month; MA, meglumine antimoniate; mg, milligrams; MI, miltefosine administered orally; microalb, microalbumin; NGAL, lipocalin-2; n.a., not applicable; neg., negative; O, observation; PB, peripheral blood; PCR, polymerase chain reaction; PON-1, paraoxonase-1; pos., positive; q12h, every 12 h; q24h, every 24 h; rbc, red blood cells; Ref, reference; SC, subcutaneous administration; T, treatment; tp, total protein; UP/C, urine protein/creatinine ratio; USG, urine specific gravity; w, week; wbc, white blood cells; w/o, without; y, year; γ-GT, gamma-glutamyltransferase; bili, bilirubin; γ, gamma; *, statistically significant; dark green fields, meglumine antimoniate monotherapy; light green fields, meglumine antimoniate combined with allopurinol; dark blue fields, miltefosine monotherapy; light blue fields, miltefosine combined with allopurinol.

#### 3.2.2. Adverse Effects

Miltefosine is slowly metabolized in the liver and mainly excreted biliary with feces, whereas it is barely detectable in the urine of treated dogs [106,130]. The main adverse effects of treatment are appetite loss, nausea, vomiting, and diarrhea, which can be mitigated by co-administration of food [124,133,135,138]. In a multicentric clinical trial, adverse effects probably related to miltefosine treatment (2 mg/kg, q24h, PO, for 28 days) were recorded for 11/94 dogs and the most frequent signs included vomiting and diarrhea. In some dogs, depression and reduced appetite up to anorexia were recorded. However, treatment did not need to be interrupted in any dog due to drug intolerance [124]. Occurrence of gastrointestinal adverse effects was not reduced by an alternative dosing regimen with lower doses of miltefosine (1.2 mg/kg, q24h, PO, for 5 days, followed by 2.5 mg/kg, q24h, PO, for 25 days) which was applied in combination with allopurinol (10 mg/kg, q12h, PO, for 180 days) to 16/34 dogs with leishmaniosis and compared to 18/34 dogs with combined treatment including regular miltefosine dosage [137].

Besides gastrointestinal signs, single cases of arrythmia, coprophagia, worsening of keratitis, osteoarthritis, polydipsia/polyuria, and a reduction in red and white blood cells (7 days after miltefosine initiation) were observed in treated dogs in clinical studies [88,124,135].

Due to potentially teratogenic, fetotoxic, and embryotoxic effects, which were proven in rats, miltefosine should not be used in pregnant and lactating bitches [122,130]. In dogs, nephrotoxic effects are not described for miltefosine. In a study on four healthy beagle dogs that received miltefosine treatment (2 mg/kg, q24h, PO) for 28 days, no renal lesions were detected by light and electron microscopy in biopsy specimens taken 28 days after end of treatment. However, increased urine protein levels were observed at the end of treatment in two out of four miltefosine-treated dogs (as well as in the meglumine antimoniate group) [106]. In a retrospective study, the effect of treatment with miltefosine (2 mg/kg, q24h, PO, for 28 days) in combination with allopurinol (10 mg/kg, q12h, PO) on proteinuria was investigated in 20 dogs. Before treatment onset, 9/20 dogs had no, 7/20 dogs had borderline, and 4/20 dogs had proteinuria. After the 28-day treatment period, 12/20 dogs had no, 7/20 dogs had borderline, and 1/20 dogs had proteinuria. UP/C ratios decreased significantly, whereas no significant differences were observed in serum creatinine and urea levels after treatment [12].

#### 3.2.3. Drug Resistance Potential

Since miltefosine is used in human medicine, the development and spread of miltefosine resistant *Leishmania* strains is a special concern [141]. Only sparse data are available for dogs so far. In an in vitro study, *Leishmania* isolated from a dog in Brazil (1) before treatment with miltefosine (2 mg/kg, q24h, PO, for 28 days) and allopurinol (10 mg/kg/day, PO), (2) in a 4-month period between two miltefosine cycles in which only allopurinol was applied, and (3) after a second miltefosine treatment cycle, showed a decrease in miltefosine susceptibility between the different timepoints. Although the dog was only treated with miltefosine and allopurinol, cross-resistance to amphotericin B was observed in the isolates [142]. Thus, repeated miltefosine cycles might be a risk factor for drug resistance, but further studies are necessary.

### 3.3. Conclusions on Leishmanicidal Treatment

Meglumine antimoniate was considered the gold standard leishmanicidal treatment in dogs with leishmaniosis for a long time. However, with the approval of miltefosine, an alternative treatment became available that was expected to have comparable efficacy on *Leishmania* parasites, without impairing kidney function and that can be administered orally [106]. Indeed, nowadays, both drugs are widely used to treat dogs with leishmaniosis, but there is no clear consensus whether one of them should be preferred over the other. Due to a synergistic effect with allopurinol, miltefosine and meglumine antimoniate are usually combined with allopurinol.

Meglumine antimoniate is commonly administered at 100 mg/kg, q24h, by subcutaneous injections for 28 days. Prolongation of the treatment period for a further 2–3 weeks is empirically advised by current guidelines in dogs with poor improvement [2], but controlled studies are missing. Nephrotoxic effects are the main limiting factor, which is why kidney function needs to be thoroughly evaluated before, during, and after treatment with meglumine antimoniate. Its parenteral administration can cause local reactions on the injection site and requires considerable effort on some pet owners (daily injections at home or at the veterinarian), which potentially compromises treatment success by improper administration or premature withdrawal of treatment [143].

Miltefosine is used as suspension for oral administration in dogs with leishmaniosis at a dose of 2 mg/kg, q24h, PO, for 28 days. Due to its pharmacokinetic properties, efficacy of treatment lasts longer than the 28-day application period. Adverse effects on gastrointestinal tract (nausea, vomiting, and diarrhea) can occur during treatment.

Comparative studies between meglumine antimoniate and miltefosine (with and without concomitant allopurinol treatment) have shown similar anti-*Leishmania* and clinical efficacy [88,91]. In dogs treated with meglumine antimoniate, the effect of treatment on disease signs and acute phase proteins might occur more rapidly than with miltefosine treatment, likely due to differences in pharmacokinetic behavior [70,93]. During a 6-year observation period, lower relapse rates were observed after treatment with meglumine antimoniate (one out of nine dogs) compared to miltefosine (four out of nine dogs) [70]. In order to draw valid conclusions on long-term outcomes, further comparative studies should be performed in non-endemic areas without risk of re-infection and a higher number of dogs. Regarding adverse effects, meglumine antimoniate has been shown to cause renal tubular damage in contrast to miltefosine [106].

When choosing a drug to treat leishmaniosis, the individual situation of each dog must always be considered and the patient’s condition/comorbidities (e.g., bleeding tendency, gastrointestinal disorders, kidney disease) have to be taken into account. According to the authors’ opinion, if dogs have never been treated with leishmanicidal agents before, miltefosine might be preferred over meglumine antimoniate due to the simple oral administration, which might be associated with better owner compliance and lower caregiver burden. Furthermore, miltefosine has a lower potential for severe adverse effects (especially important for dogs that already suffer from kidney disease). If repeated cycles of leishmanicidal treatment are indicated (relapse or insufficient improvement), change of the leishmanicidal agent in order to obtain higher efficacy and minimize risk of drug resistance seems reasonable (Table 3, Figure 2).

## 4. Outlook

There is a lack of standardization in studies on the treatment of canine leishmaniosis, which makes inter-study comparisons difficult. In the past, only a few studies have been conducted under randomized, blinded, and/or (placebo-) controlled conditions. Differences exist in the study design, e.g., mode of infection (experimental vs. natural), pre-treatment, dosing schemes, recording/scoring of disease signs, treatment success criteria, length of follow-up, different risk of re-infection (endemic vs. non-endemic areas), and (often only a small) number of included dogs. Due to the different forms of manifestation and the variety of signs in canine leishmaniosis, a validated scoring system should be developed and used uniformly in future (treatment) studies. Furthermore, studies are necessary to investigate and define treatment termination criteria, length and dosing regimens, and adaptation of treatment in dogs with individual conditions/comorbidities, resulting in recommendations for dogs infected with *L. infantum* in endemic, as well as non-endemic areas.

Especially for allopurinol, there is no consensus about treatment decision, duration, and discontinuation, and less information on individual factors influencing urinary stone formation and the actual efficacy of counteracting measures (low-purine diet, urine pH monitoring, and dose reduction).

In addition, studies on drug resistance mechanisms (leishmanistatic and leishmanicidal agents) are required to identify critical drivers for the development of drug resistance by treatment duration, (maximum) repetition cycles, and inter-treatment intervals as part of a one-health approach.

Finally, caregiver burden among owners of dogs with *Leishmania* infections should be analyzed more in detail, as this could considerably influence treatment success and outcome.

## Figures and Tables

**Table 3:** Authors’ conclusions on treatment options for dogs with *Leishmania* infections.

Drug	Indication	Main Adverse Effects	Remarks	Dose	Length of Treatment
**allopurinol** **(AL)**	signs of disease (with MA or MI) previous signs of disease (maintenance) no signs of disease (in non-endemic areas)	xanthine urolithiasis	only with low-purine diet no combination with purine containing drugs ultrasonographic screening for urinary tract mineralization	10 mg/kg, q12h, PO consider dose adjustment in case of xanthine mineralization or renal disease	at least 6 months, thereafter withdrawal only in case of remission and marked decrease in antibody levels (or no antibodies)
**meglumine** **antimoniate** **(MA)**	signs of disease (combined with AL)	nephrotoxicity injection-site reactions (pancreatitis)	caution in dogs with renal disease, bleeding tendency good owner compliance required	100 mg/kg, q24h or 50 mg/kg, q12h, SC or IV consider dose adjustment in case of renal disease	28 days, prolongation (2–3 weeks) possible in severe cases
**miltefosine** **(MI)**	signs of disease (combined with AL)	gastrointestinal signs	application together with food no use in pregnant, lactating, breeding dogs	2 mg/kg, q24h, PO	28 days, therapeutic effect beyond application period

IV, intravenous administration; kg, kilograms; mg, milligrams, q12h, every 12 h; PO, peroral administration; q24h, every 24 h; SC, subcutaneous administration.

## Data Availability

No new data were created in this study.
